# LinearTurboFold: Linear-time global prediction of conserved structures for RNA homologs with applications to SARS-CoV-2

**DOI:** 10.1073/pnas.2116269118

**Published:** 2021-12-09

**Authors:** Sizhen Li, He Zhang, Liang Zhang, Kaibo Liu, Boxiang Liu, David H. Mathews, Liang Huang

**Affiliations:** ^a^School of Electrical Engineering & Computer Science, Oregon State University, Corvallis, OR 97331;; ^b^Baidu Research, Sunnyvale, CA 94089;; ^c^Department of Biochemistry & Biophysics, University of Rochester Medical Center, Rochester, NY 14642;; ^d^Center for RNA Biology, University of Rochester Medical Center, Rochester, NY 14642;; ^e^Department of Biostatistics & Computational Biology, University of Rochester Medical Center, Rochester, NY 14642

**Keywords:** RNA secondary structure, homologous folding, conserved structures, structural alignment, SARS-CoV-2

## Abstract

The constant emergence of COVID-19 variants reduces the effectiveness of existing vaccines and test kits. Therefore, it is critical to identify conserved structures in severe acute respiratory syndrome coronavirus 2 (SARS-CoV-2) genomes as potential targets for variant-proof diagnostics and therapeutics. However, the algorithms to predict these conserved structures, which simultaneously fold and align multiple RNA homologs, scale at best cubically with sequence length and are thus infeasible for coronaviruses, which possess the longest genomes (∼30,000 nt) among RNA viruses. As a result, existing efforts on modeling SARS-CoV-2 structures resort to single-sequence folding as well as local folding methods with short window sizes, which inevitably neglect long-range interactions that are crucial in RNA functions. Here we present LinearTurboFold, an efficient algorithm for folding RNA homologs that scales linearly with sequence length, enabling unprecedented global structural analysis on SARS-CoV-2. Surprisingly, on a group of SARS-CoV-2 and SARS-related genomes, LinearTurboFold’s purely in silico prediction not only is close to experimentally guided models for local structures, but also goes far beyond them by capturing the end-to-end pairs between 5${}^{\prime }$ and 3${}^{\prime }$ untranslated regions (UTRs) (∼29,800 nt apart) that match perfectly with a purely experimental work. Furthermore, LinearTurboFold identifies undiscovered conserved structures and conserved accessible regions as potential targets for designing efficient and mutation-insensitive small-molecule drugs, antisense oligonucleotides, small interfering RNAs (siRNAs), CRISPR-Cas13 guide RNAs, and RT-PCR primers. LinearTurboFold is a general technique that can also be applied to other RNA viruses and full-length genome studies and will be a useful tool in fighting the current and future pandemics.

## Significance

Conserved RNA structures are critical for designing diagnostic and therapeutic tools for many diseases including COVID-19. However, existing algorithms are much too slow to model the global structures of full-length RNA viral genomes. We present LinearTurboFold, a linear-time algorithm that is orders of magnitude faster, making it, to our knowledge, the first method to simultaneously fold and align whole genomes of severe acute respiratory syndrome coronavirus 2 (SARS-CoV-2) variants, the longest known RNA virus (∼30 kb). Our work enables unprecedented global structural analysis and captures long-range interactions that are out of reach for existing algorithms but crucial for RNA functions. LinearTurboFold is a general technique for full-length genome studies and can help fight the current and future pandemics.

RNA plays important roles in many cellular processes (1, 2). To maintain their functions, secondary structures of RNA homologs are conserved across evolution (3–5). These conserved structures provide critical targets for diagnostics and treatments. Thus, there is a need for developing fast and accurate computational methods to identify structurally conserved regions.

Commonly, conserved structures involve compensatory base pair changes, where two positions in primary sequences mutate across evolution and still conserve a base pair; for instance, an AU or a CG pair replaces a GC pair in homologous sequences. These compensatory changes provide strong evidence for evolutionarily conserved structures (6–10). Meanwhile, they make it harder to align sequences when structures are unknown. Initially, the process of determining a conserved structure, termed comparative sequence analysis, was manual and required substantial insight to identify the conserved structure. A notable early achievement was the determination of the conserved transfer RNA (tRNA) secondary structure (11). Comparative analysis was also demonstrated to be 97% accurate compared to crystal structures for ribosomal RNAs, where the models were refined carefully over time (12).

To automate comparative analysis, three distinct algorithmic approaches were developed (13, 14). The first, “joint fold-and-align” method, seeks to simultaneously predict structures and a structural alignment for two or more sequences. This was first proposed by Sankoff (15) using a dynamic programming algorithm. The major limitation of this approach is that the algorithm runs in $O(n^{3k})$ against *k* sequences with the average sequence length *n*. Several software packages provide implementations of the Sankoff algorithm (16–21) that use simplifications to reduce runtime. The second, “align-then-fold” approach, is to input a sequence alignment and predict the conserved structure that can be identified across sequences in the alignment. This was described by Waterman (22) and was subsequently refined and popularized by RNAalifold (23). The third, “fold-then-align” approach, is to predict plausible structures for the sequences and then align the structures to determine the sequence alignment and the optimal conserved structures. This was described by Waterman (24) and implemented in RNAforester (25) and MARNA (26) (*SI Appendix*, Fig. S1).

As an alternative, TurboFold II (27), an extension of TurboFold (28), provides a more computationally efficient method to align and fold sequences. Taking multiple unaligned sequences as input, TurboFold II iteratively refines alignments and structure predictions so that they conform more closely to each other and converge on conserved structures. TurboFold II is significantly more accurate than other methods (16, 18, 23, 29, 30) when tested on RNA families with known structures and alignments.

**Fig. 1.**
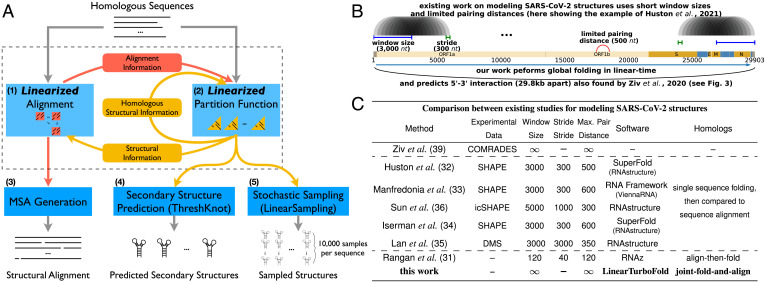
(*A*) The LinearTurboFold framework. Like TurboFold II, LinearTurboFold takes multiple unaligned homologous sequences as input and outputs a secondary structure for each sequence and a multiple-sequence alignment (MSA). But unlike TurboFold II, LinearTurboFold employs two linearizations to ensure linear runtime: a linearized alignment computation (module 1) to predict posterior coincidence probabilities (red squares) for all pairs of sequences (first four sections in *Methods*) and a linearized partition function computation (module 2) to estimate base-pairing probabilities (yellow triangles) for all the sequences (*Methods*, *Extrinsic Information Calculation* and *Methods*, *LinearPartition for Base Pairing Probabilities Estimation with Extrinsic Information*). These two modules take advantage of information from each other and iteratively refine predictions (*SI Appendix*, Fig. S2). After several iterations, module 3 generates the final multiple-sequence alignments (*Methods*, *MSA Generation and Secondary Structure Prediction*), and module 4 predicts secondary structures. Module 5 can stochastically sample structures. (*B* and *C*) Prior studies (31–36) [except for the purely experimental work by Ziv et al. (39)] used local folding methods with limited window size and maximum pairing distance. *B* shows the local folding of the SARS-CoV-2 genome by Huston et al. (32), which used a window of 3,000 nt that was advanced 300 nt. It also limited the distance between nucleotides that can form base pair at 500. Some studies also used homologous sequences to identify conserved structures (32–36), but they predicted only structures for one genome and utilized sequence alignments to identify mutations. By contrast, LinearTurboFold is a global folding method without any limitations on sequence length or paring distance, and it jointly folds and aligns homologs to obtain conserved structures. Consequently, LinearTurboFold can capture long-range interactions even across the whole genome (the long arc in *B* and Fig. 3).

However, the cubic runtime and quadratic memory usage of TurboFold II prevent it from scaling to longer sequences such as full-length severe acute respiratory syndrome coronavirus 2 (SARS-CoV-2) genomes, which contain ∼30,000 nucleotides; in fact, no joint-align-and-fold methods can scale to these genomes, which are the longest among RNA viruses. As a (not very principled) workaround, most existing efforts for modeling SARS-CoV-2 structures (31–36) resort to local folding methods (37, 38) with sliding windows plus a limited pairing distance, abandoning all long-range interactions, and only consider one SARS-CoV-2 genome (Fig. 1 *B* and *C*), ignoring signals available in multiple homologous sequences. To address this challenge, we designed a linearized version of TurboFold II, LinearTurboFold (Fig. 1*A*), which is a global homologous folding algorithm that scales linearly with sequence length. This linear runtime makes it, to our knowledge, the first joint-fold-and-align algorithm scale to full-length coronavirus genomes without any constraints on window size or pairing distance, taking about 13 h to analyze a group of 25 SARS-CoV homologs. It also leads to significant improvement on secondary structure prediction accuracy as well as an alignment accuracy comparable to or higher than all benchmarks.

Over a group of 25 SARS-CoV-2 and SARS-related homologous genomes, LinearTurboFold predictions are close to the canonical structures (40) and structures modeled with the aid of experimental data (32–34) for several well-studied regions. Due to global rather than local folding, LinearTurboFold discovers a long-range interaction involving 5${}^{\prime }$ and 3${}^{\prime }$ untranslated regions (UTRs) (∼29,800 nt apart), which is consistent with recent purely experimental work (39) and yet is out of reach for local folding methods used by existing studies (Fig. 1 *B* and *C*). In short, our in silico method of folding multiple homologs can achieve results similar to, and sometimes more accurate than, those of experimentally guided models for one genome. Moreover, LinearTurboFold identifies conserved structures supported by compensatory mutations, which are potential targets for small-molecule drugs (41) and antisense oligonucleotides (ASOs) (36). We further identify regions that are 1) sequence-level conserved; 2) at least 15 nt long; and 3) accessible (i.e., likely to be completely unpaired) as potential targets for ASOs (42), small interfering RNA (siRNA) (43), CRISPR-Cas13 guide RNA (gRNA) (44), and RT-PCR primers (45). LinearTurboFold is a general technique that can also be applied to other RNA viruses (e.g., influenza, Ebola, HIV, Zika, etc.) and full-length genome studies.

## Results

The framework of LinearTurboFold has two major aspects (Fig. 1*A*): linearized structure-aware pairwise alignment estimation (module 1) and linearized homolog-aware structure prediction (module 2). LinearTurboFold iteratively refines alignments and structure predictions, specifically, updating pairwise alignment probabilities by incorporating predicted base-pairing probabilities (from module 2) to form structural alignments and modifying base-pairing probabilities for each sequence by integrating the structural information from homologous sequences via the estimated alignment probabilities (from module 1) to detect conserved structures. After several iterations, LinearTurboFold generates the final multiple-sequence alignment (MSA) based on the latest pairwise alignment probabilities (module 3) and predicts secondary structures using the latest pairing probabilities (module 4).

LinearTurboFold achieves linear time regarding sequence length with two major linearized modules: our recent work, LinearPartition (46) (Fig. 1*A*, module 2), which approximates the RNA partition function (47) and base-pairing probabilities in linear time, and a novel algorithm, LinearAlignment (module 1). LinearAlignment aligns two sequences by a hidden Markov model (HMM) in linear time by applying the same beam search heuristic (48) used by LinearPartition. Finally, LinearTurboFold assembles the secondary structure from the final base-pairing probabilities using an accurate and linear-time method named ThreshKnot (49) (module 4).

LinearTurboFold also integrates a linear-time stochastic sampling algorithm named LinearSampling (50) (module 5), which independently samples structures according to the homolog-aware partition functions and then calculates the probability of being unpaired for regions, which is an important property in, for example, siRNA sequence design (43). Therefore, the overall end-to-end runtime of LinearTurboFold scales linearly with sequence length (first seven sections of *Methods*). The number of iterations and other hyperparameters were tuned on the training set. As observed previously (27, 28), improvements after three iterations are negligible, and therefore the best number of iterations is set to be three. On the testing set, it is observed that LinearTurboFold achieves the most substantial improvements in both structure prediction and MSA accuracies in the first iteration and continues to benefit from the next two iterations (*SI Appendix*, Fig. S5), which is consistent with the observation on the training set. After approximately three iterations, both structure prediction and MSA accuracies remain stable with small fluctuations. To better demonstrate the improvement in each iteration, we visualized both base-pairing probabilities and alignment coincidence probabilities from LinearTurboFold for a group of five tRNAs across iterations (*SI Appendix*, Figs. S6 and S7).

### Scalability and Accuracy

**Fig. 2.**
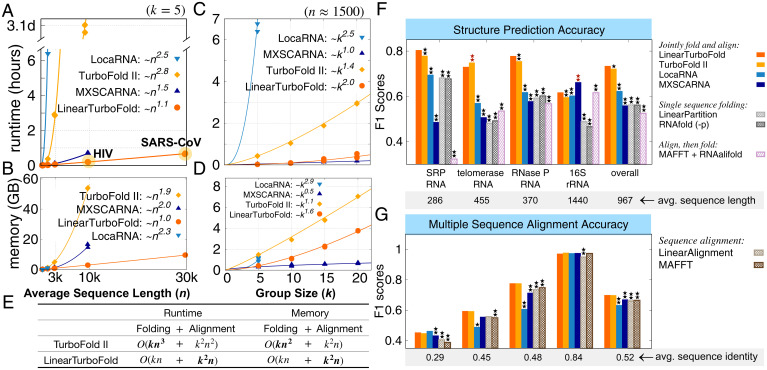
End-to-end scalability and accuracy comparisons. (*A* and *B*) End-to-end runtime and memory usage comparisons between benchmarks and LinearTurboFold against the sequence length. LinearTurboFold uses beam size 100 in both partition function and HMM alignment calculation with three iterations to run all groups of data. (*C* and *D*) End-to-end runtime and memory usage comparisons against the group size. To our knowledge, LinearTurboFold is the first joint-fold-and-align algorithm that scales to full-length coronavirus genomes (∼30,000 nt) due to its linear runtime. (*E*) The runtime and space complexity comparisons between TurboFold II and LinearTurboFold. The dominating terms are in boldface type. (*F* and *G*) The F1 accuracy scores of the structure prediction and multiple-sequence alignment (*SI Appendix*, Table S1). LocARNA and MXSCARNA are Sankoff-style simultaneous folding and alignment algorithms for homologous sequences. As negative controls, LinearPartition and Vienna RNAfold predicted structures for each sequence separately; LinearAlignment and MAFFT generated sequence-level alignments; RNAalifold folded prealigned sequences (e.g., from MAFFT) and predicted conserved structures. Statistical significances (two-tailed permutation test) between the benchmarks and LinearTurboFold are marked with one star (⋆) on the top of the corresponding bars if *P* < 0.05 or two stars $\left(\begin{array}{c} ⋆\\ ⋆ \end{array}\right)$ if *P* < 0.01. The benchmarks whose accuracies are significantly lower than LinearTurboFold are annotated with black stars, while benchmarks higher than LinearTurboFold are marked with dark red stars. Overall, on structure prediction, LinearTurboFold achieves significantly higher accuracy than all evaluated benchmarks, and on multiple-sequence alignment it achieves accuracies comparable to TurboFold II and significantly higher than other methods (*SI Appendix*, Table S1).

To evaluate the efficiency of LinearTurboFold against the sequence length, we collected a dataset consisting of seven families of RNAs with sequence length ranging from 210 to 30,000 nt, including five families from the RNAStrAlign dataset (27) plus 23S ribosomal RNA, HIV genomes, and SARS-CoV genomes, and the calculation for each family uses five homologous sequences (*Methods*, *Efficiency and Scalability Datasets*). Fig. 2*A* compares the running time of LinearTurboFold with TurboFold II and two Sankoff-style simultaneous folding and alignment algorithms, LocARNA (local alignment of RNA) and MXSCARNA. Clearly, LinearTurboFold scales linearly with sequence length *n* and is substantially faster than other algorithms, which scale superlinearly. The linearization in LinearTurboFold brings orders of magnitude speedup over the cubic-time TurboFold II, taking only 12 min on the HIV family (average length 9,686 nt), while TurboFold II takes 3.1 d (372× speedup). More importantly, LinearTurboFold takes only 40 min on five SARS-CoV sequences while all other benchmarks fail to scale. Regarding the memory usage (Fig. 2*B*), LinearTurboFold costs linear memory space with sequence length, while other benchmarks use quadratic or more memory. In Fig. 2 *C* and *D*, we also demonstrate the runtime and memory usage against the number of homologs using sets of 16S ribosomal RNAs (rRNAs) about 1,500 nt in length. The apparent complexity of LinearTurboFold against the group size *k* is higher than that of TurboFold II because the runtime of the latter is $O(kn^{3}+k^{2}n^{2})$ and is dominated by the $O(kn^{3})$ partition function calculation, thus scaling $O(k^{1.4})$ empirically. By contrast, LinearTurboFold linearizes both partition function and alignment modules, so its overall runtime becomes $O(kn+k^{2}n)$ and is instead dominated by the $O(k^{2}n)$ alignment module, therefore scaling $O(k^{2})$ in practice. A similar analysis holds for memory usage (Fig. 2*E*).*

We next compare the accuracies of secondary structure prediction and MSA between LinearTurboFold and several benchmark methods (*Methods*, *Benchmarks*). Besides Sankoff-style LocARNA and MXSCARNA, we also consider three types of negative controls: 1) single-sequence folding (partition function based), Vienna RNAfold (38) (-p mode) and LinearPartition; 2) sequence-only alignment, MAFFT (29) and LinearAlignment (a standalone version of the alignment method developed for this work but without structural information); and 3) an align-then-fold method that predicts consensus structures from MSAs (*SI Appendix*, Fig. S1), MAFFT + RNAalifold (23).

For secondary structure prediction, LinearTurboFold, TurboFold II, and LocARNA achieve higher F1 scores than single-sequence folding methods (Vienna RNAfold and LinearPartition) (Fig. 2*F*), which demonstrates folding with homology information performs better than folding sequences separately. Overall, LinearTurboFold performs significantly better than all the other benchmarks on structure prediction. For the accuracy of MSAs (Fig. 2*G*), the structural alignments from LinearTurboFold obtain higher accuracies than sequence-only alignments (LinearAlignment and MAFFT) on all four families, especially for families with low sequence identity. On average, LinearTurboFold performs comparably with TurboFold II and significantly better than other benchmarks on alignments. We also note that the structure prediction accuracy of the align-then-fold approach (MAFFT + RNAalifold) depends heavily on the alignment accuracy and is the worst when the sequence identity is low (e.g., signal recognition particle [SRP] RNA) and the best when the sequence identity is high (e.g., 16S rRNA) (Fig. 2 *F* and *G*).

### Highly Conserved Structures in SARS-CoV-2 and SARS-Related BetaCoronaviruses

RNA sequences with conserved secondary structures play vital biological roles and provide potential targets. The current COVID-19 outbreak raises an emergent requirement of identifying potential targets for diagnostics and therapeutics. Given the strong scalability and high accuracy, we used LinearTurboFold on a group of full-length SARS-CoV-2 and SARS-related (SARSr) genomes to obtain global structures and identify highly conserved structural regions.

We used a greedy algorithm to select the 16 most diverse genomes from all the valid SARS-CoV-2 genomes submitted to the Global Initiative on Sharing Avian Influenza Data (GISAID) (52) up to December 2020 (*Methods*, *SARS-CoV-2 Datasets*). We further extended the group by adding nine SARS-related homologous genomes (five human SARS-CoV-1 and four bat coronaviruses) (53). In total, we built a dataset of 25 full-length genomes consisting of 16 SARS-CoV-2 and 9 SARS-related sequences (*SI Appendix*, Fig. S9). The average pairwise sequence identities of the 16 SARS-CoV-2 and the total 25 genomes are 99.9% and 89.6%, respectively. LinearTurboFold takes about 13 h and 43 GB on the 25 genomes.

**Fig. 3.**
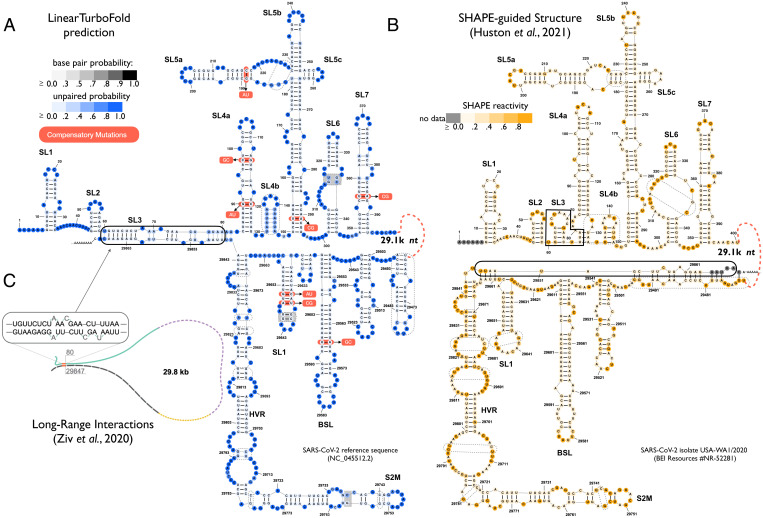
Secondary structure predictions of SARS-CoV-2 extended 5${}^{\prime }$ and 3${}^{\prime }$ UTRs. (*A*) LinearTurboFold prediction. The nucleotides and base pairs are colored by unpaired probabilities and base-pairing probabilities, respectively. The compensatory mutations extracted by LinearTurboFold are annotated with alternative pairs in red boxes (see *SI Appendix*, Table S2 for more fully conserved pairs with covariational changes). (*B*) SHAPE-guided model by Huston et al. (32) (window size 3,000 nt sliding by 300 nt with maximum pairing distance 500 nt). The nucleotides are colored by SHAPE reactivities. Dashed boxes enclose the different structures between *A* and *B*. Our model is close to Huston et al.’s (32), but the major difference is that LinearTurboFold predicts the end-to-end pairs involving 5${}^{\prime }$ and 3${}^{\prime }$ UTRs (solid box in *A*), which is exactly the same interaction detected by Ziv et al. (39) using the COMRADES experimental technique (*C*). Such long-range interactions cannot be captured by the local folding methods used by prior experimentally guided models (Fig. 1*B*). The similarity between models *A* and *B* and the exact agreement between *A* and *C* show that our in silico method of folding multiple homologs can achieve results similar to, if not more accurate than, experimentally guided single-genome prediction. As negative controls (*SI Appendix*, Fig. S10), the align-then-fold (RNAalifold) method cannot predict such long-range interactions. Although the single-sequence folding algorithm (LinearPartition) predicts a long-range 5${}^{\prime }$–3${}^{\prime }$ interaction, the positions are not the same as the LinearTurboFold model and Ziv et al.’s (39) experimental result.

To evaluate the reliability of LinearTurboFold predictions, we first compare them with Huston et al.’s (32) SHAPE-guided models for regions with well-characterized structures across betacoronaviruses. For the extended 5${}^{\prime }$ and 3${}^{\prime }$ UTRs, LinearTurboFold’s predictions are close to the SHAPE-guided structures (Fig. 3 *A* and *B*), i.e., both identify the stem loops (SLs) 1 to 2 and 4 to 7 in the extended 5${}^{\prime }$ UTR and the bulged stem loop (BSL), SL1, and a long bulge stem for the hypervariable region (HVR) including the stem-loop II-like motif (S2M) in the 3${}^{\prime }$ UTR. Interestingly, in our model, the high unpaired probability of the stem in the SL4b indicates the possibility of being single stranded as an alternative structure, which is supported by experimental studies (33, 36). In addition, the compensatory mutations LinearTurboFold found in UTRs strongly support the evolutionary conservation of structures (Fig. 3*A*).

The most important difference between LinearTurboFold’s prediction and Huston et al.’s (32) experimentally guided model is that LinearTurboFold discovers an end-to-end interaction (29.8 kb apart) between the 5${}^{\prime }$ UTR (SL3, 60 to 82 nt) and the 3${}^{\prime }$ UTR (final region, 29,845 to 29,868 nt), which fold locally by themselves in Huston et al.’s (32) model. Interestingly, this 5${}^{\prime }$–3${}^{\prime }$ interaction matches exactly with the one discovered by the purely experimental work of Ziv et al. (39) using the COMRADES technique to capture long-range base-pairing interactions (Fig. 3*C*). These end-to-end interactions have been well established by theoretical and experimental studies (54–56) to be common in natural RNAs, but are far beyond the reaches of local folding methods used in existing studies on modeling SARS-CoV-2 secondary structures (32–35). By contrast, LinearTurboFold predicts secondary structures globally without any limit on window size or base-pairing distance, enabling it to discover long-distance interactions across the whole genome. The similarity between our predictions and the experimental work shows that our in silico method of folding multiple homologs can achieve results similar to, if not more accurate than, those of experimentally guided single-genome predictions.

LinearTurboFold can model these end-to-end interactions due to three ingredients: 1) linearization, 2) LinearPartition’s better modeling power on long sequences and long-range pairs, and 3) homologous folding and soft alignment. Linearization not only enables LinearTurboFold to scale to longer sequences, but also improves the accuracy of modeling long-range interactions benefiting from LinearPartition (46). In addition, homologous folding is also crucial. We observed that LinearPartition can model the same end-to-end interactions detected by Ziv et al. (39) for 8 of 25 sequences (4 of 16 SARS-CoV-2 and 4 of 9 SARS-related sequences; *SI Appendix*, Figs. S12*A* and S13, *Left* column). For the other sequences, however, LinearPartition either cannot predict end-to-end interactions or predicts them in the wrong locations. On the other hand, LinearTurboFold propagates the correct structural information from those eight sequences to other homologs, resulting in all SARS-CoV-2 sequences having the same end-to-end pairs (*SI Appendix*, Figs. S12*B* and S13, *Right* column). By contrast, the align- then-fold approach (MAFFT + RNAalifold), which relies on the input hard alignment and predicts one single consensus structure for all homologs, fails to predict such long-range interactions (*SI Appendix*, Fig.S10*B*).

**Fig. 4.**
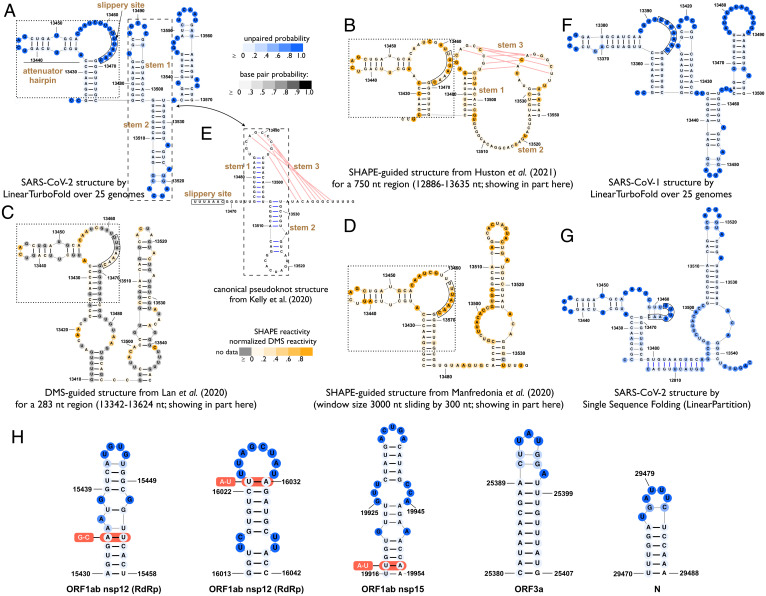
(*A*–*D*) Secondary structure predictions of SARS-CoV-2 extended frameshifting stimulation element (FSE) region (13,425 to 13,545 nt). (*A*) LinearTurboFold prediction. (*B*–*D*) Experimentally guided predictions from the literature (32, 33, 35), which are sensitive to the context and region boundaries due to the use of local folding methods (*SI Appendix*, Fig. S11). (*E*) The canonical pseudoknot structure by the comparative analysis between SARS-CoV-1 and SARS-CoV-2 genomes (40). For the 5${}^{\prime }$ region of the FSE shown in dotted boxes (attenuator hairpin, internal loop with slippery site, and a stem), the LinearTurboFold prediction (*A*) is consistent with *B*–*D*; for the 3${}^{\prime }$ region of the FSE shown in dashed boxes, our prediction (predicting stems 1 to 2 but missing stem 3) is closer to the canonical structure in *E* compared to *B*–*D*. (*F*) LinearTurboFold prediction on SARS-CoV-1. (*G*) Single-sequence folding algorithm (LinearPartition) prediction on SARS-CoV-2, which is quite different from LinearTurboFold’s. As another negative control, the align-then-fold method (RNAalifold) predicts a rather dissimilar structure (*SI Appendix*, Fig. S11*G*). (*H*) Five examples from 59 fully conserved structures among 25 genomes (*SI Appendix*, Table S3), 26 of which are different compared with prior work (31, 32).

**Fig. 5.**
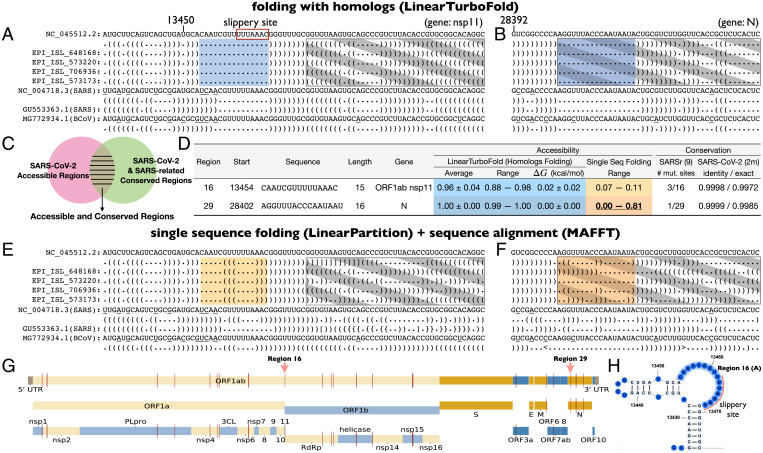
An illustration of accessible and conserved regions that LinearTurboFold identifies. (*A* and *B*) Identified structurally conserved accessible regions by LinearTurboFold with the help of considering alignment and folding simultaneously. The regions at least 15 nt long with accessibility of at least 0.5 among all the 16 SARS-CoV-2 genomes are shaded on blue background. Structures are encoded in dot-bracket notation. “(” and “)” indicate nucleotides pairing in the 3${}^{\prime }$ and 5${}^{\prime }$ directions, respectively. “.” indicates an unpaired nucleotide. The positions with mutations compared to the SARS-CoV-2 reference sequence among three different subfamilies (SARS-CoV-2, SARS-CoV-1, and BCoV) are underlined. (*C*) Accessible and conserved regions are not only accessible among SARS-CoV-2 genomes (pink circle) but also conserved (at sequence level) among both SARS-CoV-2 and SARS-related genomes (green circle). (*D*) Two examples of 33 accessible and conserved regions found by LinearTurboFold. Regions 16 and 29 correspond to the accessible regions in *A* and *B*, respectively. Region 16 is also the long internal loop including the slippery site in the FSE region (*H*). The conservation of these regions on nine SARS-related genomes is the number of mutated sites. The conservation on the ∼2 million SARS-CoV-2 dataset is shown in both average sequence identity with the reference sequence and the percentage of exact matches, respectively. (*E* and *F*) Single-sequence folding algorithms predict greatly different structures even if the sequence identities are high (gray rectangles with diagonal strips). These two regions, fully conserved among SARS-CoV-2 genomes, still fold into different structures due to mutations outside the regions. By contrast, LinearTurboFold folds all sequences to the same structures due to the homologous signals in the corresponding regions in *A* and *B*. (*G*) The positions of these 33 regions (red bars) across the whole genome (*SI Appendix*, Table S5). All the accessible and conserved regions are potential targets for siRNAs, ASOs, CRISPR-Cas13 gRNAs, and RT-PCR primers.

The frameshifting stimulation element (FSE) is another well-characterized region. For an extended FSE region, the LinearTurboFold prediction consists of two substructures (Fig. 4*A*): The 5${}^{\prime }$ part includes an attenuator hairpin and a stem, which are connected by a long internal loop (16 nt) including the slippery site, and the 3${}^{\prime }$ part includes three stem loops. We observe that our predicted structure of the 5${}^{\prime }$ part is consistent with that in experimentally guided models (32, 33, 35) (Fig. 4 *B*–*D*). In the attenuator hairpin, the small internal loop motif (UU) was previously selected as a small-molecule binder that stabilizes the folded state of the attenuator hairpin and impairs frameshifting (41). For the long internal loop including the slippery site, we show in the next section that it is both highly accessible and conserved (Fig. 5), which makes it a perfect candidate for drug design. For the 3${}^{\prime }$ region of the FSE, LinearTurboFold successfully predicts stems 1 to 2 (but misses stem 3) of the canonical three-stem pseudoknot (40) (Fig. 4*E*). Our prediction is closer to the canonical structure compared to that in the experimentally guided models (32, 33, 35) (Fig. 4 *B*–*D*); one such model (Fig. 4*B*) identified the pseudoknot (stem 3) but with an open stem 2. Note that all these experimentally guided models for the FSE region were estimated for specific local regions. As a result, the models are sensitive to the context and region boundaries (32, 35, 57) (see *SI Appendix*, Fig.S11*D*–*F* for alternative structures of Fig. 4 *B*–*D* with different regions). LinearTurboFold, by contrast, does not suffer from this problem by virtue of global folding without local windows. Besides SARS-CoV-2, we note that the estimated structure of the SARS-CoV-1 reference sequence (Fig. 4*F*) from LinearTurboFold is similar to SARS-CoV-2 (Fig. 4*A*), which is consistent with the observation that the structure of the FSE region is highly conserved among betacoronaviruses (40). Finally, as negative controls, both the single-sequence folding algorithm (LinearPartition in Fig. 4*G*) and the align-then-fold method (RNAalifold in *SI Appendix*, Fig.S11*G*) predict quite different structures compared with the LinearTurboFold prediction (Fig. 4*A*) (39/61% of pairs from the LinearTurboFold model are not found by LinearPartition/RNAalifold).

In addition to the well-studied UTRs and FSE regions, LinearTurboFold discovers 50 conserved structures with identical structures among 25 genomes, and 26 regions are different compared to previous studies (31, 32) (Fig. 4*H* and *SI Appendix*, Table S3). These different structures are potential targets for small-molecule drugs (41) and ASOs (36, 58). LinearTurboFold also recovers fully conserved base pairs with compensatory mutations (*SI Appendix*, Table S2), which implies highly conserved structural regions whose functions might not have been explored. We provide the complete multiple-sequence alignment and predicted structures for 25 genomes from LinearTurboFold (Dataset S1; see *SI Appendix*, Fig. S14 for the format).

### Highly Accessible and Conserved Regions in SARS-CoV-2 and SARS- Related Betacoronaviruses

Studies show that the siRNA silencing efficiency, ASO inhibitory efficacy, CRISPR-Cas13 knockdown efficiency, and RT-PCR primer binding efficiency all correlate with the target region’s accessibility (43–45, 59), which is the probability of a target site being fully unpaired. However, most existing work for designing siRNAs, ASOs, CRISPR-Cas13 gRNAs, and RT-PCR primers does not take this feature into consideration (60, 61) (*SI Appendix*, Table S4). Here, LinearTurboFold is able to provide more principled design candidates by identifying accessible regions of the target genome. In addition to accessibility, the emerging variants around the world reduce effectiveness of existing vaccines and test kits (*SI Appendix*, Table S4), which indicates sequence conservation is another critical aspect for therapeutic and diagnostic design. LinearTurboFold, being a tool for both structural alignment and homologous folding, can identify regions that are both (sequence-wise) conserved and (structurally) accessible, and it takes advantage of not only SARS-CoV-2 variants but also homologous sequences, e.g., SARS-CoV-1 and bat coronavirus genomes, to identify conserved regions from historical and evolutionary perspectives.

To get unstructured regions, Rangan et al. (31) imposed a threshold on unpaired probability of each position, which is a crude approximation because the probabilities are not independent of each other. By contrast, the widely used stochastic sampling algorithm (50, 62) builds a representative ensemble of structures by sampling independent secondary structures according to their probabilities in the Boltzmann distribution. Thus, the accessibility for a region can be approximated as the fraction of sampled structures in which the region is single stranded. LinearTurboFold utilized LinearSampling (50) to generate 10,000 independent structures for each genome according to the modified partition functions after the iterative refinement (Fig. 1*A*, module 5) and calculated accessibilities for regions at least 15 nt long. We then define accessible regions that are with at least 0.5 accessibility among all 16 SARS-CoV-2 genomes (Fig. 5 *A* and *B*). We also measure the free energy to open a target region [i,j] (63), notated $\Delta G_{\text{u}}[i,j]=-RT(\log\;Z_{\text{u}}[i,j]-\log\;Z)=-RT\log\;P_{\text{u}}[i,j]$, where *Z* is the partition function that sums up the equilibrium constants of all possible secondary structures, $Z_{\text{u}}[i,j]$ is the partition function over all structures in which the region [i,j] is fully unpaired, *R* is the universal gas constant, and *T* is the thermodynamic temperature. Therefore $P_{\text{u}}[i,j]$ is the unpaired probability of the target region and can be approximated via sampling by $s_{0}/s$, where *s* is the sample size and *s*_0_ is the number of samples in which the target region is single stranded. The regions whose free-energy changes are close to zero need less free energy to open and are thus more accessible to bind with siRNAs, ASOs, CRISPR-Cas13 gRNAs, and RT-PCR primers.

Next, to identify conserved regions that are highly conserved among both SARS-CoV-2 and SARS-related genomes, we require that these regions contain at most three mutated sites on the nine SARS-related genomes compared to the SARS-CoV-2 reference sequence because historically conserved sites are also unlikely to change in the future (64), and the average sequence identity with reference sequence over a large SARS-CoV-2 dataset is at least 0.999 (here we use a dataset of ∼2 million SARS-CoV-2 genomes submitted to GISAID up to 30 June 2021^†^; *Methods*, *SARS-CoV-2 Datasets*). Finally, we identified 33 accessible and conserved regions (Fig. 5*G* and *SI Appendix*, Table S5), which are not only structurally accessible among SARS-CoV-2 genomes but also highly conserved among SARS-CoV-2 and SARS-related genomes (Fig. 5*C*). Because the specificity is also a key factor influencing siRNA efficiency (65), we used BLAST against the human transcript dataset to search for these regions (*SI Appendix*, Table S5). Finally, we also listed the GC content of each region. Among these regions, region 16 corresponds to the internal loop containing the slippery site in the extended FSE region, and it is conserved at both structural and sequence levels (Fig. 5 *D* and *H*). Besides SARS-CoV-2 genomes, the SARS-related genomes such as the SARS-CoV-1 reference sequence (NC_004718.3) and a bat coronavirus (BCoV) (MG772934.1) also form similar structures around the slippery site (Fig. 5*A*). By removing the constraint of conservation on SARS-related genomes, we identified 38 additional candidate regions (*SI Appendix*, Table S6) that are accessible but only highly conserved on SARS-CoV-2 variants.

We also designed a negative control by analyzing the SARS-CoV-2 reference sequence alone using LinearSampling, which can also predict accessible regions. However, these regions are not structurally conserved among the other 15 SARS-CoV-2 genomes, resulting in vastly different accessibilities, except for one region in the M gene (*SI Appendix*, Table S7). The reason for this difference is that, even with a high sequence identity (over 99.9%), single-sequence folding algorithms still predict greatly dissimilar structures for the SARS-CoV-2 genomes (Fig. 5 *E* and *F*). Both regions (in nsp11 and N genes) are fully conserved among the 16 SARS-CoV-2 genomes, yet they still fold into vastly different structures due to mutations outside the regions; as a result, the accessibilities are either low (nsp11) or in a wide range (N) (Fig. 5*D*). Conversely, addressing this by folding each sequence with proclivity of base pairing inferred from all homologous sequences, LinearTurboFold structure predictions are more consistent with each other and thus can detect conserved structures (Fig. 5 *A* and *B*).

## Discussion

The constant emergence of new SARS-CoV-2 variants is reducing the effectiveness of exiting vaccines and test kits. To cope with this issue, there is an urgent need to identify conserved structures as promising targets for therapeutics and diagnostics that would work despite current and future mutations. Here we presented LinearTurboFold, an end-to-end linear-time algorithm for structural alignment and conserved structure prediction of RNA homologs, which is, to our knowledge, the first joint-fold-and-align algorithm that scales to full-length SARS-CoV-2 genomes without imposing any constraints on base-pairing distance. We also demonstrate that LinearTurboFold leads to significant improvement on secondary structure prediction accuracy as well as an alignment accuracy comparable to or higher than all benchmarks.

Unlike existing work on SARS-CoV-2 using local folding and single-sequence folding workarounds, LinearTurboFold enables unprecedented global structural analysis on SARS-CoV-2 genomes; in particular, it can capture long-range interactions, especially the one between 5${}^{\prime }$ and 3${}^{\prime }$ UTRs across the whole genome, which matches perfectly with a recent purely experimental work. Over a group of SARS-CoV-2 and SARS-related homologs, LinearTurboFold identifies not only conserved structures supported by compensatory mutations and experimental studies, but also accessible and conserved regions as vital targets for designing efficient small-molecule drugs, siRNAs, ASOs, CRISPR-Cas13 gRNAs, and RT-PCR primers. LinearTurboFold is widely applicable to the analysis of other RNA viruses (influenza, Ebola, HIV, Zika, etc.) and full-length genome analysis.

## Methods

### Pairwise Hidden Markov Model

We use a pairwise hidden Markov model (pair-HMM) to align two sequences (51, 66). The model includes three actions (*h*): aligning two nucleotides from two sequences (ALN), inserting a nucleotide in the first sequence without a corresponding nucleotide in the other sequence (INS1), and a nucleotide insertion in the second sequence without a corresponding nucleotide in the first sequence (INS2). We then define A(x,y) as a set of all the possible alignments for the two sequences and one alignment a∈A(x,y) as a sequence of steps (*h*, *i*, *j*) with *m* + 2 steps, where (*h*, *i*, *j*) means an alignment step at the position pair (*i*, *j*) by the action *h*. Thus, for the *l*th step $a_{l}=(h_{l},i_{l},j_{l})\in a$, the values of *i_l_* and *j_l_* depend on the action *h_l_* and the positions $i_{l-1}$ and $j_{l-1}$ of $a_{l-1}$:

$$a_{l}=\{\begin{array}{cccc} (\mathrm{ALN}, & i_{l-1}+1, & j_{l-1}+1), & h_{l}=\mathrm{ALN}\\ (\mathrm{INS1}, & i_{l-1}+1, & j_{l-1}), & h_{l}=\mathrm{INS1}\\ (\mathrm{INS2}, & i_{l-1}, & j_{l-1}+1), & h_{l}=\mathrm{INS2} \end{array}$$

with (ALN,0,0) as the first step and (ALN,|x|+1,|y|+1) as the last one. For two sequences {ACAAGU, AACUG}, one possible alignment {–ACAAGU, AAC–UG} can be specified as {(ALN,0,0)→(INS2,0,1)→(ALN, 1,2)→(ALN,2,3)→(INS1,3,3)→ (INS1,4,3)→(ALN,5,4)→(ALN,6,5)→ (ALN,7,6)}, where a gap symbol (–) represents a nucleotide insertion in the other sequence at the corresponding position (*SI Appendix*, Fig. S3). The action *h_l_* in each step $\left(h_{l},i_{l},j_{l}\right)$ corresponds to a line segment starting from the previous node $\left(i_{l-1},j_{l-1}\right)$ and stopping at the node (*i_l_*, *j_l_*). Thus, the line segment is horizontal, vertical, or diagonal toward the top-right corner when *h_l_* is INS1, INS2, or ALN, respectively (*SI Appendix*, Fig. S3).

We initialize the first step with the state ALN of probability 1; thus $p_{\pi }(\mathrm{ALN})=1$. $p_{\text{t}}(h_{2}|h_{1})$ is the transition probability from the state *h*_1_ to *h*_2_, and $p_{\text{e}}((c_{1},c_{2})|h_{1})$ is the probability of the state *h*_1_ emitting a character pair (*c*_1_, *c*_2_) with values from {A, G, C, U, –}. Both the emission and transition probabilities were taken from TurboFold II. The function e() yields a character pair based on *a_l_* and the nucleotides of two sequences:

$$e(x,y,a_{l})=\{\begin{array}{cc} (x_{i_{l}},y_{j_{l}}), & h_{l}=\mathrm{ALN}\\ (x_{i_{l}},-), & h_{l}=\mathrm{INS1}\\ (-,y_{j_{l}}), & h_{l}=\mathrm{INS2} \end{array},$$

where *x_i_* and *y_j_* are the *i*th and *j*th nucleotides of sequences **x** and **y**, respectively. Note that the first step $a_{0}=(\mathrm{ALN},0,0)$ and the last $a_{m+1}=(\mathrm{ALN},|x|+1,|y|+1)$ do not have emissions.

We denote forward probability $\alpha_{i,j}^{h}$ encompassing the probability of the partial alignments of **x** and **y** up to positions *i* and *j* and all the alignments that go through the step (*h*, *i*, *j*):

$$\begin{array}{ll} \alpha_{i,j}^{h} & =\sum_{\underset{\exists k,a_{k}=(h,i,j)}{a\in A(x,y)}}p(x,y,a[:k])\\ & =p_{\pi }(h_{0})\cdot \prod_{l=1}^{k}p_{\text{t}}(h_{l}|h_{l-1})p_{\text{e}}(e(x,y,a_{l})|h_{l}), \end{array}$$

where a[:k] indicates the partial alignments from the starting node up to the *k*th step and $a_{k}=(h,i,j)$. For instance, $\alpha_{3,3}^{\mathrm{ALN}},\;\alpha_{3,3}^{\mathrm{INS1}}$, and $\alpha_{3,3}^{\mathrm{INS2}}$ correspond to the region circled by the blue dashed lines (*SI Appendix*, Fig. S3*B*–*D*). Similarly, the backward probability $\beta_{i,j}^{h}$ assembles the probability of partial alignments a[k+1:] from the (k+1) th step up to the end one:

$$\begin{array}{ll} \beta_{i,j}^{h} & =\sum_{\underset{\exists k,a_{k}=(h,i,j)}{a\in A(x,y)}}p(x,y,a[k+1:])\\ & =\left\{\prod_{l=k+1}^{m}p_{\text{t}}(h_{l}|h_{l-1})p_{\text{e}}(e(x,y,a_{l})|h_{l})\right\}\cdot p_{\text{t}}(h_{m+1}|h_{m}). \end{array}$$

For example, $\beta_{3,3}^{\mathrm{ALN}},\;\beta_{3,3}^{\mathrm{INS1}}$, and $\beta_{3,3}^{\mathrm{INS2}}$ are the regions circled by the yellow dashed line (*SI Appendix*, Fig. 3 *B*–*D*). Thus, the probability of observing two sequences p(x,y) is $\alpha_{|x|+1,|y|+1}^{\mathrm{ALN}}$ or $\beta_{0,0}^{\mathrm{ALN}}$.

### Posterior Coincidence Probability Computation

Nucleotide positions *i* and *j* in two sequences **x** and **y** are said to be coincident (notated as i∼j) in an alignment *a* if the alignment path goes through the node (*i*, *j*) (51). Since the node (*i*, *j*) is reachable by three actions H={ALN,INS1,INS2}, the coincidence probability for a position pair (*i*, *j*) given two sequences is

[1] $$p(i∼j|x,y)=\frac{1}{p(x,y)}\sum_{\underset{\exists h,(h,i,j)\in a}{a\in A(x,y)}}p(x,y,a),$$

where p(x,y,a) is the probability of two sequences with the alignment *a*, and p(x,y) is the probability of observing two sequences, which is the sum of probability of all the possible alignments:

$$p(x,y)=\sum_{a\in A(x,y)}p(x,y,a).$$

The coincidence probability for positions *i* and *j* (Eq. **1**) can be computed by

$$p(i∼j|x,y)=\frac{\sum_{h}\alpha_{i,j}^{h}\cdot \beta_{i,j}^{h}}{\alpha_{|x|+1,|y|+1}^{\mathrm{ALN}}}.$$

### LinearAlignment

Unlike a previous method (51) that fills out all the nodes in the alignment matrix by columns (*SI Appendix*, Fig. S3), LinearAlignment scans the matrix based on the step count *s*, which is the sum value of *i* and *j* (s=i+j) for the partial alignments of $x_{\left[1,i\right]}$ and $y_{\left[1,j\right]}$. As shown in the pseudocode (*SI Appendix*, Fig. S4), the forward phase starts from the node (0, 0) in the state ALN of probability 1 and then iterates the step count *s* from 0 to |x|+|y|−1. For each step count *s* with a specific state *h* from H, we first collect all the nodes (*i*, *j*) with the step count *s* with $\alpha_{i,j}^{h}$ existing, which means the position pair (*i*, *j*) has been visited via the state *h* before. Then each node makes transitions to next nodes by their states and updates the corresponding forward probabilities $\alpha_{i+1,j}^{\mathrm{INS1}},\;\alpha_{i,j+1}^{\mathrm{INS2}}$, and $\alpha_{i+1,j+1}^{\mathrm{ALN}}$, respectively.

The current alignment algorithm is still an exhaustive-search algorithm and costs quadratic time and space for all the |x|×|y| nodes. To reduce the runtime, LinearAlignment uses the beam search heuristic algorithm (48) and keeps a limited number of promising nodes at each step. For each step count *s* with a state *h*, LinearAlignment applies the beam search method first over *B*(*s*, *h*), which is the collection of all the nodes (*i*, *j*) with step count *s* and the presence of $\alpha_{i,j}^{h}$ (*SI Appendix*, Fig. S4, line 6). This algorithm saves only the top $b_{\text{align}}$ nodes with the highest forward scores in *B*(*s*, *h*), and these are subsequently allowed to make transitions to the next states. Here $b_{\text{align}}$ is a user-specified beam size and the default value is 100. In total, $O(b_{\text{align}}n)$ nodes survive because the length of *s* is |x|+|y| and each step count keeps $b_{\text{align}}$ nodes. For simplicity, we show the topological order and the beam search method with alignment examples (*SI Appendix*, Fig.S3*A*), while the forward–backward algorithm adopts the same idea by summing the probabilities of all the possible alignments.

After the forward phase, the backward phase (*SI Appendix*, Fig. S4) performs in linear time to calculate the coincidence probabilities automatically because only a linear number of nodes in *B*(*s*, *h*) are stored. Thus by pruning low-scoring candidates at each step in the forward algorithm, we reduce the runtime from $O(n^{2})$ to $O(b_{\text{align}}n)$ for aligning two sequences. For *k* input homologous sequences, LinearTurboFold computes posterior coincidence probabilities for each pair of sequences by LinearAlignment, which costs $O(k^{2}b_{\text{align}}n)$ runtime in total.

### Match Scores Computation and Modified LinearAlignment

To encourage the pairwise alignment conforming with estimated secondary structures, LinearTurboFold predicts structural alignments by incorporating the secondary structural conformation. PMcomp (67) first proposed the match score to measure the structural similarity for position pairs between a pair of sequences, and TurboFold II adapts it as a prior. Based on the base pair probabilities $P_{x}(i,j)$ estimated from the partition function for a sequence **x**, a position *i* could be paired with bases upstream or downstream or unpaired, with corresponding probability $P_{x,>}(i)=\sum_{j<i}P_{x}(i,j),\;P_{x,<}(i)=\sum_{j>i}P_{x}(i,j)$, and $P_{x,o}(i)=1-P_{x,>}(i)-P_{x,<}(i)$, respectively. The match score $m_{x,y}(i,j)$ for two positions *i* and *j* from two sequences **x** and **y** is based on the probabilities of these three structural propensities from the last iteration (*t* –1):

$$\begin{array}{ll} m_{x,y}^{\left(t\right)}(i,j) & =\alpha_{1}\left[\sqrt{P_{x,>}^{\left(t-1\right)}(i)\cdot P_{y,>}^{\left(t-1\right)}(j)}\sqrt{P_{x,<}^{\left(t-1\right)}(i)\cdot P_{y,<}^{\left(t-1\right)}(j)}\right]\\ & \;+\alpha_{2}\sqrt{P_{x,o}^{\left(t-1\right)}(i)\cdot P_{y,o}^{\left(t-1\right)}(j)}+\alpha_{3}, \end{array}$$

where *α*_1_, *α*_2_, and *α*_3_ are weight parameters trained in TurboFold II. The forward–backward phrases integrate the match score as a prior when aligning two nucleotides (*SI Appendix*, Fig. S4, lines 10 and 12).

TurboFold II separately precomputes match scores for all the $O(n^{2})$ position pairs for pairs of sequences before the HMM alignment calculation. However, only a linear number of pairs $O(b_{\text{align}}n)$ survive after applying the beam pruning in LinearAlignment. To reduce redundant time and space usage, LinearTurboFold calculates the corresponding match scores for coincident pairs when they are first visited in LinearAlignment. Overall, for *k* homologous sequences, LinearTurboFold reduces the runtime of the whole module of pairwise posterior coincidence probability computation from $O(k^{2}n^{2})$ to $O(k^{2}b_{\text{align}}n)$ by applying the beam search heuristic to the pairwise HMM alignment and calculating only the match scores for position pairs that are needed.

### Extrinsic Information Calculation

To update partition functions for each sequence with the structural information from homologs, TurboFold (28) introduces extrinsic information to model the proclivity for base pairing induced from the other sequences in the input set S. The extrinsic information $e_{x}(i,j)$ for a base pair (*i*, *j*) in the sequence **x** maps the estimated base-pairing probabilities of other sequences to the target sequence via the coincident nucleotides between each pair of sequences:

$$\sum_{y\in \{S\setminus x\}}\left(1-s_{x,y}\right)\sum_{k,l}p_{y}^{\left(t-1\right)}(k,l)\cdot p_{x,y}^{\left(t\right)}(i∼k)\cdot p_{x,y}^{\left(t\right)}(j∼l),$$

where $p_{y}^{\left(t-1\right)}(k,l)$ is the base pair probability for a base pair (*k*, *l*) in the sequence **y** from the (t−1) th iteration. $p_{x,y}^{\left(t\right)}(i∼k)$ and $p_{x,y}^{\left(t\right)}(j∼l)$ are the posterior coincidence probabilities for position pairs (*i*, *k*) and (*j*, *l*), respectively, from the (*t*) th iteration. The extrinsic information $e_{x}^{\left(t\right)}(i,j)$ first sums all the base pair probabilities of alignable pairs from another one sequence with the coincidence probabilities and then iterates over all the other sequences. $s_{x,y}$ is the sequence identity for sequences **x** and **y**. The sequences with a low identity contribute more to the extrinsic information than sequences of higher identity. The sequence identity is defined as the fraction of nucleotides that are aligned and identical in the alignment.

### LinearPartition for Base-Pairing Probabilities Estimation with Extrinsic Information

The classical partition function algorithm scales cubically with sequence length. The slowness limits its extension to longer sequences. To address this bottleneck, our recent LinearPartition (46) algorithm approximates the partition function and base-paring probability matrix computation in linear time. LinearPartition is significantly faster and correlates better with the ground-truth structures than the traditional cubic partition function calculation. Thus, LinearTurboFold uses LinearPartition to predict base pair probabilities instead of the traditional $O(n^{3})$-time partition function.

TurboFold introduces the extrinsic information $e_{x}^{\left(t\right)}(i,j)$ in the partition function as a pseudofree energy term for each base pair (*i*, *j*). Similarly, in LinearPartition, for each span [i,j], which is the subsequence $x_{i}\ldots x_{j}$, and its associated partition function *Q*(*i*, *j*), the partition function is modified as $\tilde{Q}(i,j)=Q(i,j)e_{x}^{\left(t\right)}{\left(i,j\right)}^{\lambda }$ if (*x_i_*, *x_j_*) is an allowed pair, where *λ* denotes the contribution of the extrinsic information relative to the intrinsic information. Specifically, at each step *j*, among all possible spans [i,j] where *x_i_* and *x_j_* are paired, we replace the original partition function *Q*(*i*, *j*) with $Q(i,j)e_{x}^{\left(t\right)}{\left(i,j\right)}^{\lambda }$ by multiplying the extrinsic information. Then LinearTurboFold applies the beam pruning heuristic over the modified partition function $\tilde{Q}(i,j)$ instead of the original.

Similarly, TurboFold II obtains the extrinsic information for all the $O(n^{2})$ base pairs before the partition function calculation of each sequence, while only a linear number of base pairs survives in LinearPartition. Thus, LinearTurboFold requires only the extrinsic information for those promising base pairs that are visited in LinearPartition. Overall, for *k* homologous sequences, LinearTurboFold reduces the runtime of base pair probabilities estimation for each sequence from $O(kn^{3}+k^{2}n^{2})$ to $O(kb_{\text{folding}}^{2}n+k^{2}b_{\text{align}}n)$ by applying the beam size $b_{\text{folding}}$ to the partition function calculation and calculating only extrinsic information for the saved base pairs.

### MSA Generation and Secondary Structure Prediction

After several iterations, TurboFold II builds the multiple-sequence alignment using a probabilistic consistency transformation, generating a guide tree and performing progressive alignment over the pairwise posterior coincidence probabilities (30). The whole procedure is accelerated in virtue of the sparse matrix by discarding alignment pairs of probability smaller than a threshold (0.01 by default). Since LinearAlignment uses the beam search method and saves only a linear number of coincident pairs, the MSA generation in LinearTurboFold costs linear runtime against the sequence length straightforwardly.

Estimated base pair probabilities are fed into downstream methods to predict secondary structures. To maintain the end-to-end linear-time property, LinearTurboFold uses ThreshKnot (49), which is a thresholded version of ProbKnot (68) and considers only base pairs of probability exceeding a threshold *θ* (θ=0.3 by default). We evaluate the performance of ThreshKnot and the maximum expected accuracy (MEA) structures with different hyperparameters (*θ* and *γ*). On a sampled RNAStrAlign training set, ThreshKnot is closer to the upper right hand than MEA, which indicates that ThreshKnot always has a higher sensitivity than MEA at a given positive predictive value (PPV) (*SI Appendix*, Fig. S8).

### Efficiency and Scalability Datasets

Four datasets are built and used for measuring efficiency and scalability. To evaluate the efficiency and scalability of LinearTurboFold with sequence length, we collected groups of homologous RNA sequences with sequence length ranging from 200 to 29,903 nt with a fixed group size 5. Sequences are sampled from the RNAStrAlign dataset (27), the Comparative RNA Web (CRW) site (69), the Los Alamos HIV database (https://www.hiv.lanl.gov/), and the SARS-related betacoronaviruses (SARS-related) (53). RNAStrAlign, aggregated and released with TurboFold II, is an RNA alignment and structure database. Sequences in RNAStrAlign are categorized into families, i.e., sets of homologs, and some families are further split into subfamilies. Each subfamily or family includes a multiple-sequence alignment and ground-truth structures for all the sequences. Twenty groups of five homologs were randomly chosen from the small-subunit ribosomal RNA (Alphaproteobacteria subfamily), SRP RNA (Protozoan subfamily), RNase P RNA (bacterial type A subfamily), and telomerase RNA families. For longer sequences, we sampled five groups of 23S rRNA (of sequence length ranging from 2,700 to 2,926 nt) from the CRW site, HIV-1 genetic sequences (of sequence length ranging from 9,597 to 9,738 nt) from the Los Alamos HIV database, and SARS-related sequences (of sequence length ranging from 29,484 to 29,903 nt). All the sequences in one group belong to the same subfamily or subtype. We sampled five groups for each family and obtained 35 groups in total. Due to the runtime and memory limitations, we did not run TurboFold II on SARS-CoV-2 groups (Fig. 2 *A* and *B*).

To assess the runtime and memory usage of LinearTurboFold with group size, we fixed the sequence length around 1,500 nt and sampled five groups of sequences from the small-subunit ribosomal RNA (Alphaproteobacteria subfamily) with group sizes 5, 10, 15, and 20, respectively (Fig. 2 *C* and *D*). We used a Linux machine (CentOS 7.7.1908) with a 2.30-GHz Intel Xeon E5-2695 v3 CPU and 755 GB memory and gcc 4.8.5 for benchmarks.

We built a test set from the RNAStrAlign dataset to measure and compare the performance between LinearTurboFold and other methods. Sixty groups of input sequences consisting of five homologous sequences were randomly selected from the small-subunit rRNA (Alphaproteobacteria subfamily), SRP RNA (Protozoan subfamily), RNase P RNA (bacterial type A subfamily), and telomerase RNA families from the RNAStrAlign dataset. We removed sequences shorter than 1,200 nt for the small-subunit rRNA to filter out subdomains and removed sequences that are shorter than 200 nt for SRP RNA following the TurboFold II paper to filter out less reliable sequences. We resampled the test set five times and show the average PPV, sensitivity, and F1 scores over the five samples (Fig. 2 *F* and *G*).

An RNAStrAlign training set was built to compare accuracies between MEA and ThreshKnot. Forty groups of three, five, and seven homologs were randomly sampled from 5S ribosomal RNA (Eubacteria subfamily), group I intron (IC1 subfamily), transfer-messenger RNA, and tRNA families from the RNAStrAlign dataset. We chose *θ* = 0.1, 0.2, 0.3, 0.4, and 0.5 for ThreshKnot and *γ* = 1, 1.5, 2, 2.5, 3, 3.5, 4, 8, and 16 for MEA. We reported the average secondary structure prediction accuracies (PPV and sensitivity) across all training families (*SI Appendix*, Fig.S8).

### Benchmarks

The Sankoff algorithm (15) uses dynamic programming to simultaneously fold and align two or more sequences, and it requires $O(n^{3k})$ time and $O(n^{2k})$ space for *k* input sequences with the average length *n*. Both LocARNA (16) and MXSCARNA (18) are Sankoff-style algorithms.

LocARNA costs $O(n^{2}(n^{2}+k^{2}))$ time and $O(n^{2}+k^{2})$ space by restricting the alignable regions. MXSCARNA progressively aligns multiple sequences as an extension of the pairwise alignment algorithm SCARNA (70) with improved score functions. SCARNA first aligns stem fragment candidates and then removes the inconsistent matching in the postprocessing to generate the sequence alignment. MXSCARNA reduces runtime to $O(k^{3}n^{2})$ and space to $O(k^{2}n^{2})$ with a limited searching space of folding and alignment. Both MXSCARNA and LocARNA uses precomputed base pair probabilities for each sequence as structural input. All the benchmarks use the default options and hyperparameters running on the RNAStrAlign test set. TurboFold II iterates three times and then predicts secondary structures by MEA (*γ* = 1). LinearTurboFold also runs three iterations with default beam sizes ($b_{\text{align}}=b_{\text{folding}}=100$) in LinearAlignment and LinearPartition and then predicts structures with ThreshKnot (θ=0.3).

### Significance Test

We use a paired, two-tailed permutation test (71) to measure the significant difference. Following the common practice, the repetition number is 10,000, and the significance threshold *α* is 0.05.

### SARS-CoV-2 Datasets

We used two large SARS-CoV-2 datasets. The first dataset is used to draw a representative sample of most diverse SARS-CoV-2 genomes. We downloaded all the genomes submitted to GISAID (52) by 29 December 2020 (downloaded on 29 December 2020) and filtered out low-quality genomes (with more than 5% unknown characters and degenerate bases, shorter than 29,500 nt, or with framing error in the coding region), and we also discarded genomes with more than 600 mutations compared with the SARS-CoV-2 reference sequence (NC_0405512.2) (72). After preprocessing, this dataset includes about 258,000 genomes. To identify a representative group of samples with more variable mutations, we designed a greedy algorithm to select 16 most diverse genomes found at least twice in the 258,000 genomes. The general idea of the greedy algorithm is to choose genomes one by one with the most new mutations compared with the selected samples, which consists of only the reference sequence at the beginning.

The second, larger, dataset is to evaluate the conservation of regions with respect to more up-to-date variants. We did the same preprocessing as the first dataset on all the genomes submitted to GISAID by 30 June 30 2021 (downloaded on 25 July 2021). This resulted in a dataset of ∼2 million genomes, which was used to evaluate conservation in Fig. 5 and *SI Appendix*, TablesS4–S6.

## Supplementary Material

Supplementary File

Supplementary File

## Data Availability

Our code, data, and complete results for 25 SARS-CoV-2 and SARS-related genomes are released at GitHub, https://github.com/LinearFold/LinearTurboFold, and our web server is at http://linearfold.org/linearturbofold. Previously published data were used for this work (27, 53).

## Footnotes

^*^Theoretically, the alignment part takes $O(k^{2}n^{2})$ space. However, in practice, TurboFold II discards positions whose alignment coincidence probabilities are less than thresholds and keeps only a linear number of positions (51).

^†^The average sequence identity is 0.9987 on that ∼2 million dataset (downloaded on 25 July 2021).
