# Supplementary material for: LinearTurboFold: Linear-time global prediction of conserved structures for RNA homologs with applications to SARS-CoV-2
Source: Proc Natl Acad Sci U S A. 2021 Dec 9;118(52):e2116269118. doi: 10.1073/pnas.2116269118 (PMC8719904; doi:10.1073/pnas.2116269118)
Supplement: Supplementary File [file pnas.2116269118.sapp.pdf]

PNAS
www.pnas.org

Supplementary Information for

LinearTurboFold: Linear-Time Global Prediction of Conserved Structures for RNA Homologs with Applications to SARS-CoV-2

Sizhen Li, He Zhang, Liang Zhang, Kai Zhao, Boxiang Liu, David H. Mathews*, and Liang Huang*

Liang Huang: liang.huang.sh@gmail.com; David H. Mathews: David_Mathews@urmc.rochester.edu

This PDF file includes:

Figs. S1 to S14
Tables S1 to S7
Legend for Dataset S1
SI References

Other supplementary materials for this manuscript include the following:

Dataset S1

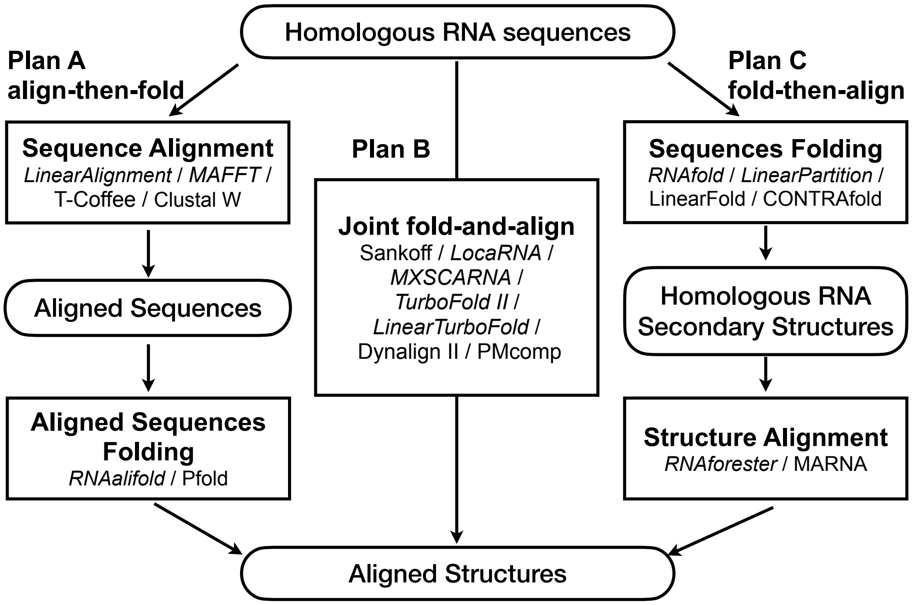

Fig. S1. Approaches of automate comparative sequence analysis can be categorized into three plans (1–3). Plan A, approach, involves two steps: first aligning sequences and then taking this alignment as input to predict the conserved structure. This was described by Waterman (4), and was subsequently refined and popularized by RNAalifold (5). This plan works well for homologs with a high sequence identity (Tab. S1). Plan C, "fold-then-align" approach, predicts plausible structures for the sequences first, and then aligns the structures to determine the sequence alignment and the optimal conserved structures. This was described by Waterman (6) and implemented in RNAforester (7) and MARNA (8). Italic methods in each plan are evaluated on RNAStrAlign dataset (Tab. S1). Plan B, "fold-and-align" approach, performs joint folding and alignment for multiple sequences, which requires more time and space. This was first proposed by Sankoff (9) using a dynamic programming algorithm. Several software packages provide implementations of the Sankoff algorithm (10–15) that use simplifications to reduce runtime.

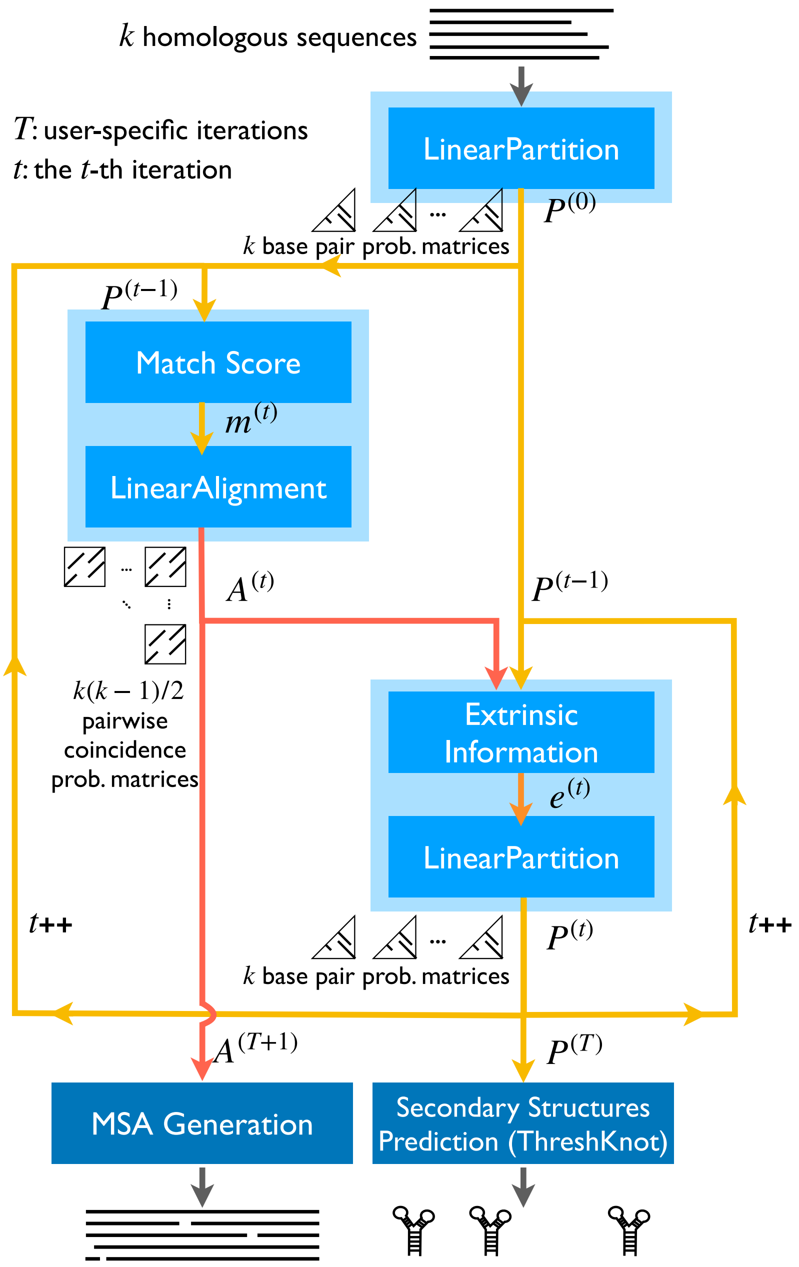

Fig. S2. The flowchart of LinearTurboFold with more detailed information. At the zeroth iteration, LinearPartition calculates the partition function and estimates the base pair probabilities for each sequence. From iteration 1 to T, the two major modules LinearAlignment and LinearPartition are conducted and updated in order with the match score and extrinsic information, respectively. The match score and extrinsic information are required and calculated for promising position pairs and base pairs during the LinearAlignment and LinearPartition computations, respectively. After T iterations, the match score and LinearAlignment computations are performed one more time over the latest the base pair probabilities. A multiple sequence is generated based on the pairwise co-incidence probabilities from the (T+1)-th iteration, and secondary structures are predicted according to the base pair probabilities for each sequence from the T-th iteration.

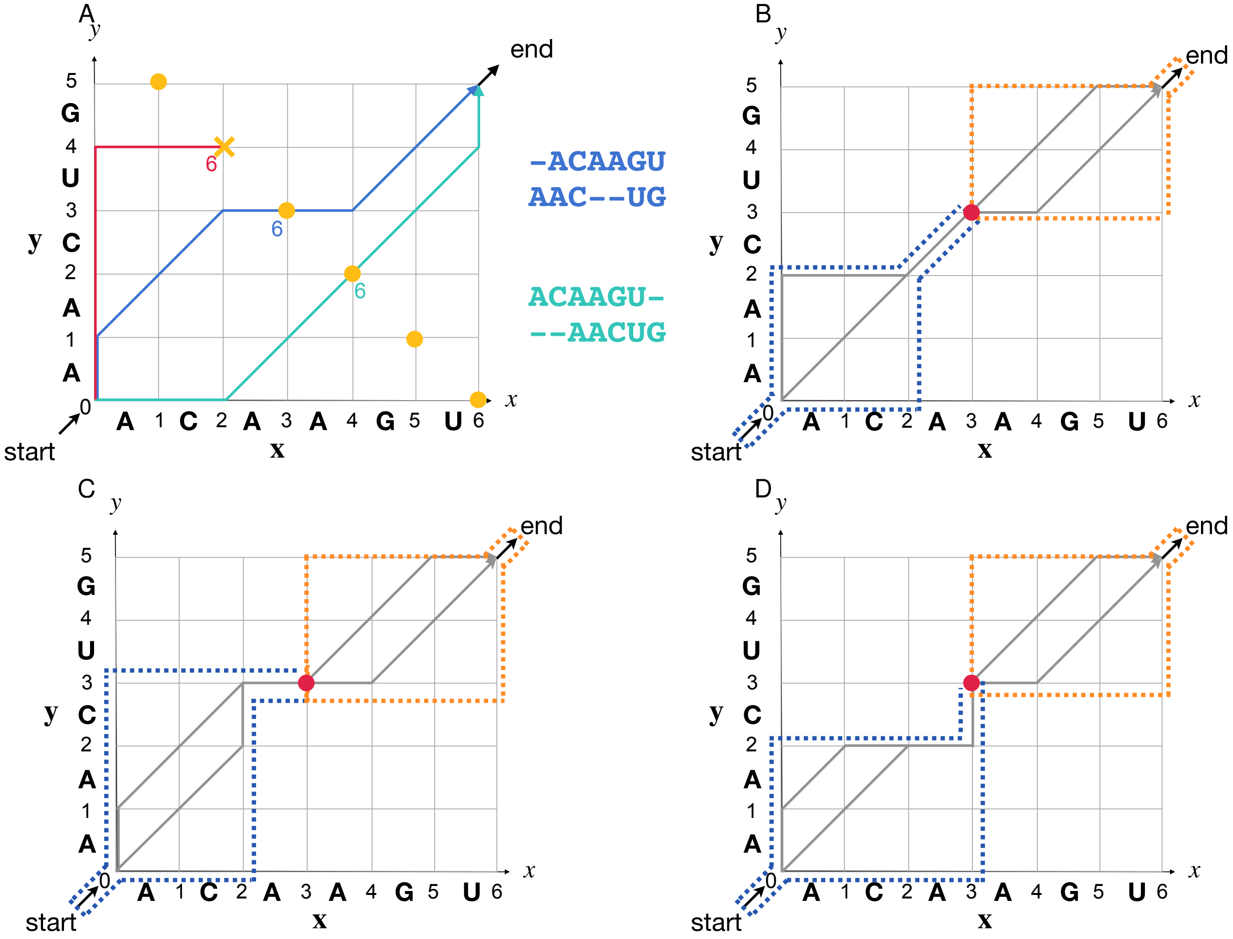

Fig. S3. Illustrations of LinearAlignment. $\mathbf{A}$: An example of aligning two sequences and the beam search method based on the step count. The x-axis and y-axis of the matrix represent two sequences $\mathbf{x}$ and $\mathbf{y}$. Yellow notes have the same step count 6. At step count 6, the red path is discarded because of its lower probability compared to others. There are two complete alignment paths (in green and blue) and the observed alignments are on the right side of the matrix with corresponding colors. $\mathbf{B}$: The area enclosed by the blue dashed line corresponds to $\alpha^{\text{ALN}}_{3,3}$, which includes all the partial alignments arriving at the node $(3, 3)$ by the state h. And the region circled by the orange dashed line maintains all the partial alignments starting from the step $(\text{ALN}, 3, 3)$ $(\beta^{\text{ALN}}_{3,3})$. $\mathbf{C}$ and $\mathbf{D}$: The regions circled by the blue dashed lines are $\alpha^{\text{INS1}}_{3,3}$ and $\alpha^{\text{INS2}}_{3,3}$, and regions circled by the orange dashed lines are $\beta^{\text{INS1}}_{3,3}$ and $\beta^{\text{INS2}}_{3,3}$, respectively.

function FORWARD($\mathbf{x}$, $\mathbf{y}$, b_{aln}) ▷ the forward phase
$\alpha^{\text{ALN}}_{0,0} \leftarrow 1$ ▷ initial probability distribution
for $s = 0 \ldots |\mathbf{x}| + |\mathbf{y}| - 1$ **do** ▷ topological order
for each h **in** $\mathcal{H}$ **do**
$B(s, h) \leftarrow$ all the nodes (i, j) such that $\alpha^h_{i,j}$ exists and $i + j = s$
BEAMPRUNE($B(s, h), b_{aln}$) ▷ keep top b_{aln} nodes in $B(s, h)$ by $\alpha^h_{i,j}$
for each node (i, j) **in** $B(s, h)$ **do** ▷ transitions to next states
$\alpha^{\text{INS1}}_{i+1,j} \mathrel{+}= \alpha^h_{i,j} \cdot p_{\text{t}}(\text{INS1} \mid h) \cdot p_{\text{e}}((x_{i+1}, -) \mid \text{INS1})$
$\alpha^{\text{INS2}}_{i,j+1} \mathrel{+}= \alpha^h_{i,j} \cdot p_{\text{t}}(\text{INS2} \mid h) \cdot p_{\text{e}}((-, y_{j+1}) \mid \text{INS2})$
$\alpha^{\text{ALN}}_{i+1,j+1} \mathrel{+}= \alpha^h_{i,j} \cdot p_{\text{t}}(\text{ALN} \mid h) \cdot p_{\text{e}}((x_{i+1}, y_{j+1}) \mid \text{ALN})$
return α

1. Pseudocode of the LinearAlignment algorithm forward phase

function BACKWARD($\mathbf{x}$, $\mathbf{y}$, α, B) ▷ the backward phase
$\beta \leftarrow \text{hash}()$
$\beta^{\text{ALN}}_{|\mathbf{x}|+1,|\mathbf{y}|+1} \leftarrow 1$ ▷ initialization
$p_{\mathbf{x},\mathbf{y}} \leftarrow \alpha^{\text{ALN}}_{|\mathbf{x}|+1,|\mathbf{y}|+1}$ ▷ probability of observing two sequences
$p_{i,j} \leftarrow 0$ ▷ co-incident probability initialization
for $s = |\mathbf{x}| + |\mathbf{y}| \ldots 0$ **do**
for each h **in** $\mathcal{H}$ **do**
for each node (i, j) **in** $B(s, h)$ **do** ▷ $B(s, h)$ saves b_{aln} entries during the forward phase
if $i = |\mathbf{x}|$ **and** $j = |\mathbf{y}|$ **then** ▷ boundary conditions
$\beta^h_{i,j} = p_{\text{t}}(\text{ALN} \mid h) \cdot \beta^{\text{ALN}}_{|\mathbf{x}|+1,|\mathbf{y}|+1}$
else
$\beta^h_{i,j} = p_{\text{t}}(\text{ALN} \mid h) \cdot p_{\text{e}}((x_{i+1}, y_{j+1}) \mid \text{ALN}) \cdot \beta^{\text{ALN}}_{i+1,j+1}$
$+ p_{\text{t}}(\text{INS1} \mid h) \cdot p_{\text{e}}((x_{i+1}, -) \mid \text{INS1}) \cdot \beta^{\text{INS1}}_{i+1,j}$
$+ p_{\text{t}}(\text{INS2} \mid h) \cdot p_{\text{e}}((-, y_{j+1}) \mid \text{INS2}) \cdot \beta^{\text{INS2}}_{i,j+1}$
$p_{i,j} \mathrel{+}= \frac{\alpha^h_{i,j} \cdot \beta^h_{i,j}}{p_{\mathbf{x},\mathbf{y}}}$ ▷ update co-incident probabilities

2. Pseudocode of the LinearAlignment algorithm backward phase (co-incidence probability computation)

Fig. S4. The pseudocode of the LinearAlignment algorithm forward and backward phases (co-incidence probability computation). The pseudocode ignores boundary conditions for simplicity. LinearAlignment applies the beam search method first over $B(s, h)$, which is the collection of all the nodes (i, j) with step count s and the presence of $\alpha^h_{i,j}$ (the line in red). This algorithm only saves the top b_{aln} nodes with the highest forward scores in $B(s, h)$, and these are subsequently allowed to make transitions to the next states. Here b_{aln} is a user-specified beam size and the default value is 100. In total, $O(b_{aln} n)$ nodes survive because the length of s is $|\mathbf{x}| + |\mathbf{y}|$ and each step count keeps b_{aln} nodes.

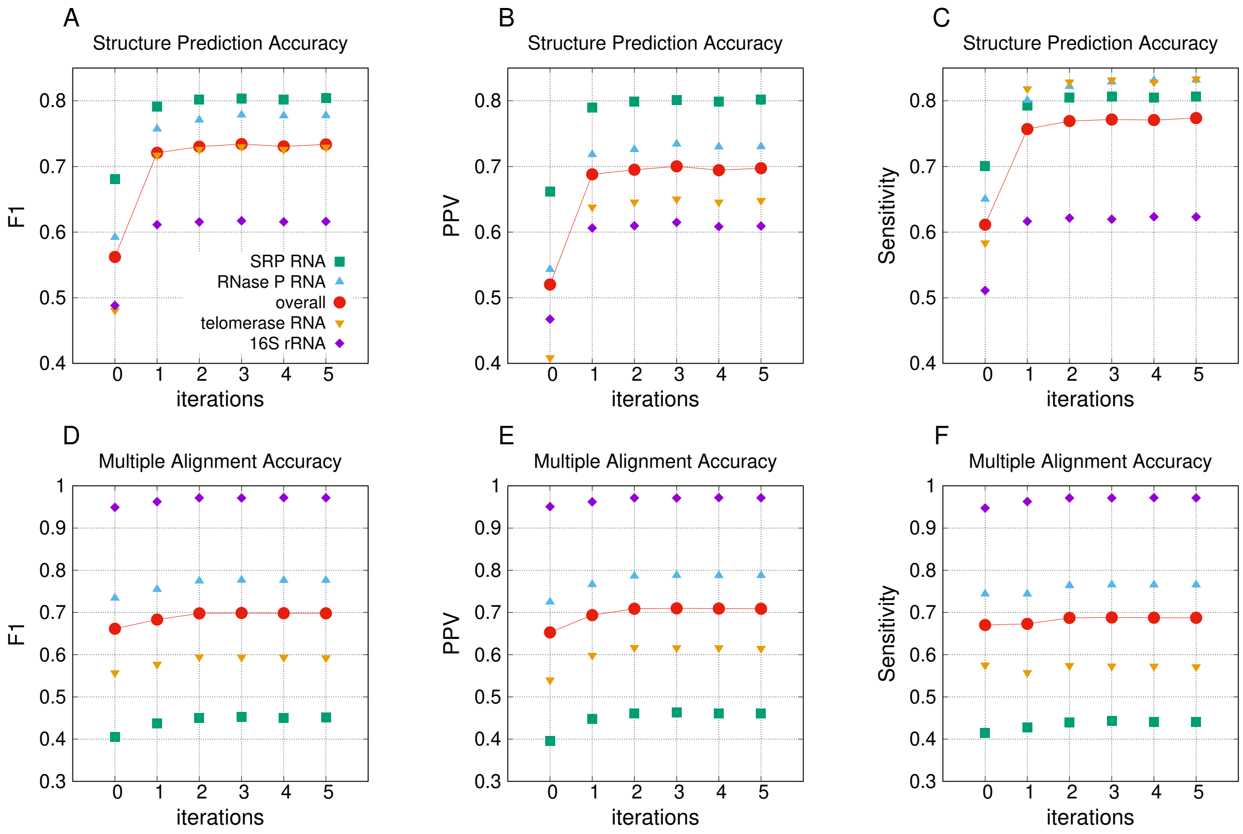

Fig. S5. Structure prediction and multiple sequence alignment accuracies against the number of iterations on the test set.

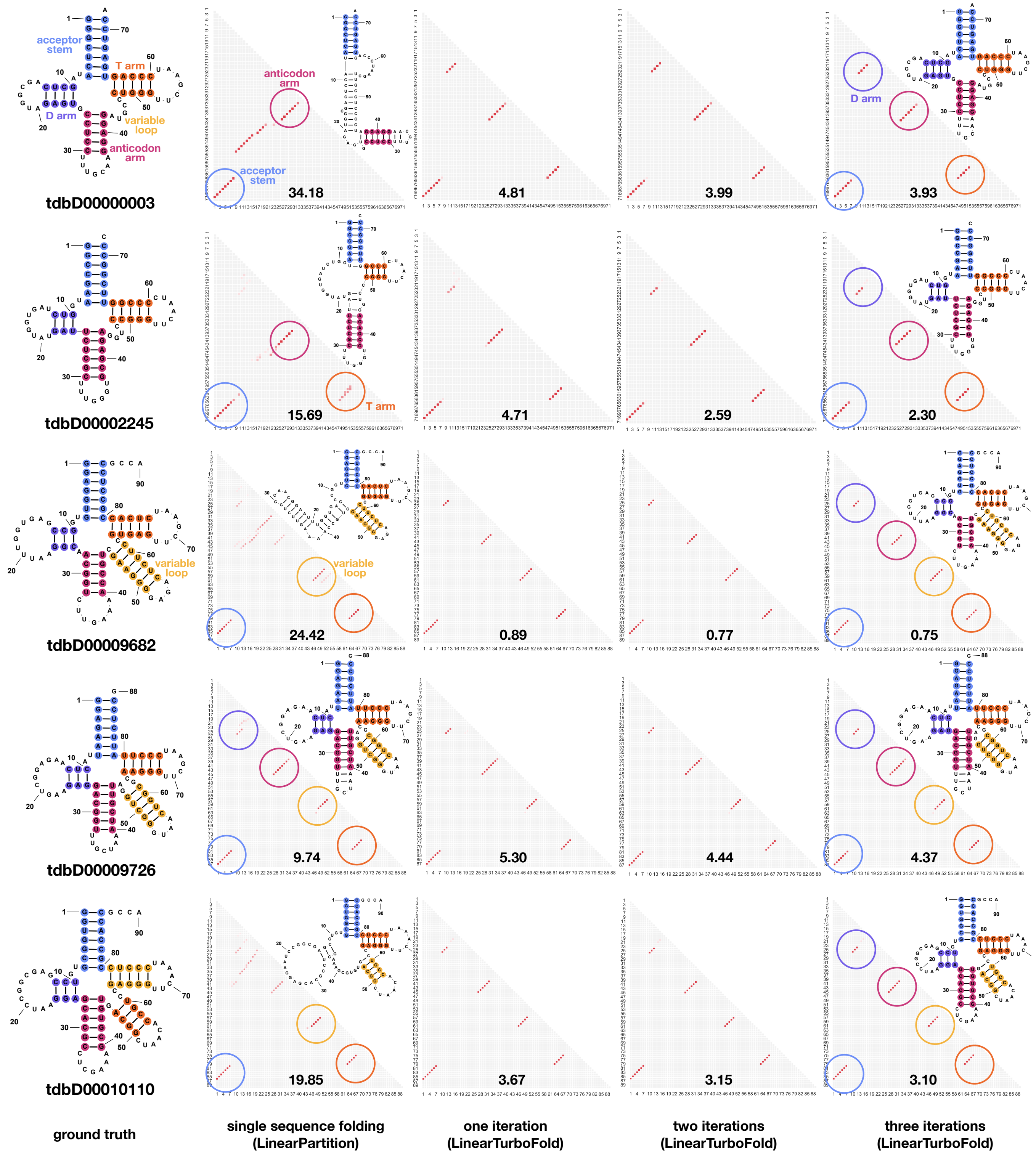

Fig. S6. Base pairing probabilities for a group of five tRNA sequences (tdbD00000003, tdbD00002245, tdbD00009682, tdbD00009726 and tdbD00010110). The first column shows the ground truth structures. The second column illustrates the base pair probabilities predicted by LinearPartition with corresponding ThreshKnot structures. The third to fifth columns show the base pair probabilities predicted by LinearTurboFold with different iterations. Ensemble defect values (16), which is the average number of incorrectly estimated paired nucleotides at equilibrium relative to the ground truth, are annotated on the bottom of lower triangles. We can observe that LinearPartition folds four sequences into wrong formations. By contrast, LinearTurboFold corrects the wrong predictions and sharpens the correct structures with larger probabilities iteratively, i.e., the ensemble defect values get smaller with more iterations. Alignment co-incidence probabilities between tdbD00002245 and tdbD00009726 across iterations are shown in Fig. S7.

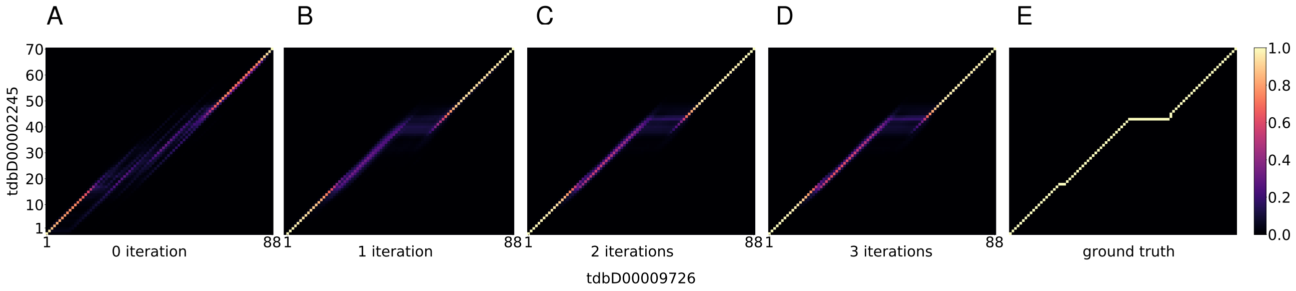

Fig. S7. Alignment co-incidence probabilities estimated by LinearTurboFold for a group of five tRNA sequences (tdbD00000003, tdbD00002245, tdbD00009726, tdbD00009682 and tdbD00010110) with different iterations. $\mathbf{A}$–$\mathbf{D}$: Alignment co-incidence probabilities between tdbD00002245 and tdbD00009726 from the zeroth iteration to the third iteration. The zeroth iteration means HMM sequence alignment without structural information. $\mathbf{E}$: The ground truth alignment. The variable loop is short and open in tdbD00002245, while a long stem loop inserted in this region in tdbD00009726 (see Fig. S6 for ground truth structures). As the iterations proceed, a long insertion is getting clearer around the variable loop in two sequences, and the probability matrix gets closer to the ground truth alignment.

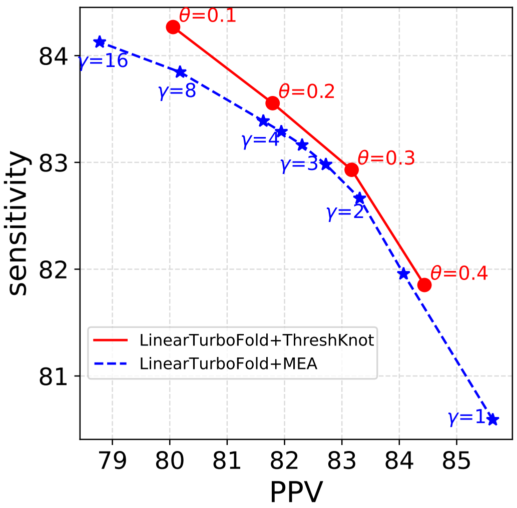

Fig. S8. Accuracy comparison between ThreshKnot and MEA on the training set with different hyper-parameters.

Fig. S9. Detailed information of the sampled 16 SARS-CoV-2 genomes and 9 SARS-related genomes. This dataset includes the reference sequences of SARS-CoV-2 and SARS-CoV-1 (NC_0405512.2, NC_004718.3). Most of the SARS-CoV-2 genomes include the D614G mutation, which has been a dominate mutation in the SARS-CoV-2 spike protein. B.1.1.7 lineage is a more infectious and lethal variant of SARS-CoV-2 first detected in the United Kingdom around November 2020. We utilized MAFFT (17) to generate the multiple sequence alignment and calculated the sequence identity with the reference sequence. The matrix under the table shows the pairwise sequence identities among 25 sequences.

Accession ID	Species	Type	Submitted Date	Location	Length	Frequency	Mutations	Sequence identity	Note
NC_045512.2	human	SARS-CoV-2	2020-01-17	Wuhan, Asia	29903	2	-	-	-
EPI_ISL_454994	human	SARS-CoV-2	2020-03-02	Wuhan, Asia	29864	3	36	0.999	
EPI_ISL_572982	human	SARS-CoV-2	2020-08-28	England, Europe	29882	2	28	0.999	D614G
EPI_ISL_573173	human	SARS-CoV-2	2020-09-08	England, Europe	29851	2	22	0.999	D614G
EPI_ISL_573220	human	SARS-CoV-2	2020-09-09	England, Europe	29784	2	19	0.999	D614G
EPI_ISL_576666	human	SARS-CoV-2	2020-09-18	England, Europe	29891	2	22	0.999	D614G
EPI_ISL_639684	human	SARS-CoV-2	2020-10-03	Latvia, Europe	29840	3	23	0.999	D614G
EPI_ISL_648168	human	SARS-CoV-2	2020-10-13	Sweden, Europe	29858	3	22	0.999	D614G
EPI_ISL_706936	human	SARS-CoV-2	2020-10-13	England, Europe	29828	2	23	0.999	D614G
EPI_ISL_638950	human	SARS-CoV-2	2020-10-14	Scotland, Europe	29891	2	29	0.999	D614G
EPI_ISL_654499	human	SARS-CoV-2	2020-10-20	Sweden, Europe	29876	3	20	0.999	D614G
EPI_ISL_666966	human	SARS-CoV-2	2020-10-30	USA, NorthAmerica	29879	2	23	0.999	D614G
EPI_ISL_704698	human	SARS-CoV-2	2020-11-01	England, Europe	29834	5	50	0.999	D614G B.1.1.7
EPI_ISL_723671	human	SARS-CoV-2	2020-11-08	England, Europe	29876	2	32	0.999	D614G
EPI_ISL_602304	human	SARS-CoV-2	2020-11-12	England, Europe	29838	2	27	0.999	D614G
EPI_ISL_710589	human	SARS-CoV-2	2020-11-19	Sweden, Europe	29815	2	28	0.999	D614G
NC_004718.3	human	SARS-CoV-1	2003-04-13	Vancouver, Canada	29751	-	6277	0.789	-
AY297028	human	SARS-CoV-1	2003-05-19	Beijing, Asia	29715	-	6306	0.788	-
AY515512.1	human	SARS-CoV-1	2005-01-01	Hong Kong, Asia	29731	-	6298	0.788	-
DQ182595.1	human	SARS-CoV-1	2005-08-26	Zhejiang, Asia	29706	-	6298	0.788	-
GU553363.1	human	SARS-CoV-1	2010-01-15	USA, NorthAmerica	29644	-	6351	0.786	-
DQ022305.2	bat	SARS-CoV-1	2005-04-29	Hong Kong, Asia	29728	-	6337	0.787	-
DQ648857.1	bat	SARS-CoV-1	2006-05-23	Hong Kong, Asia	29741	-	6285	0.789	-
EPI_ISL_402131	bat	SARS-CoV-2	2013-07-24	Yunnan, Asia	29855	-	1176	0.961	-
MG772934.1	bat	SARS-CoV-1	2008-01-05	Jiangsu, Asia	29732	-	3740	0.874	-

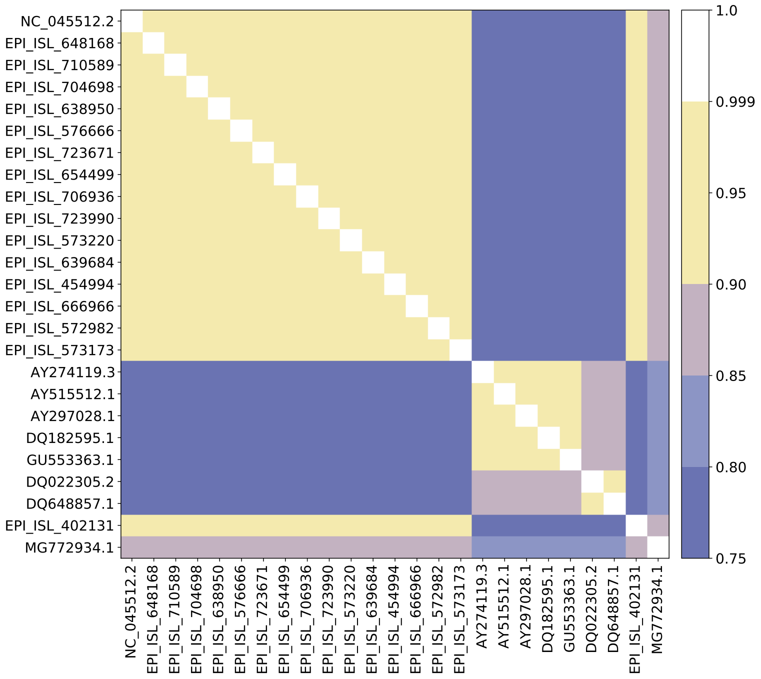

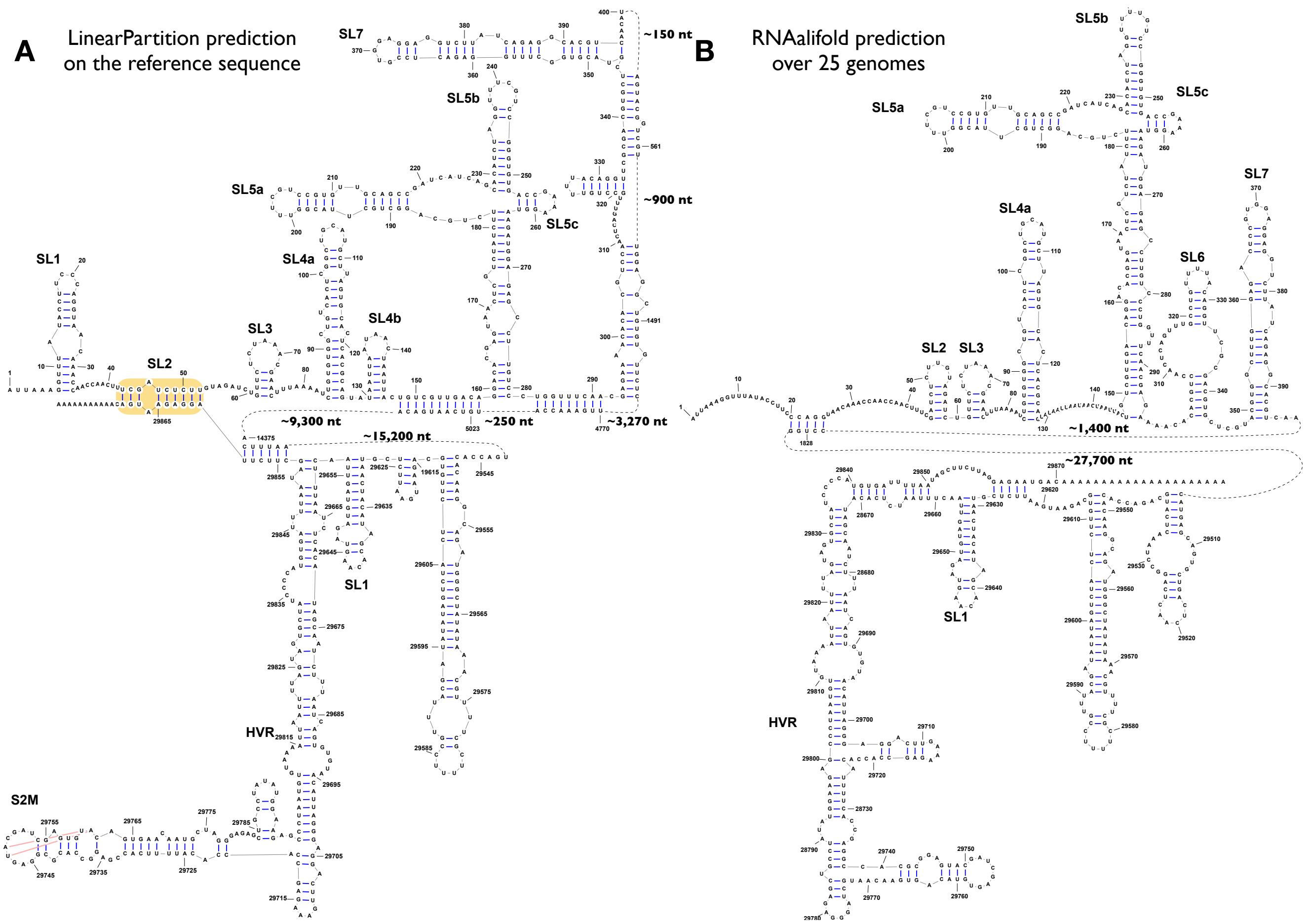

Fig. S10. Secondary structure prediction of SARS-CoV-2 for extended 5' and 3' UTRs. $\mathbf{A}$: LinearPartition prediction of the SARS-CoV-2 reference sequence (NC_0405512.2) alone (single sequence folding). LinearPartition also predicts a long-range interaction between 5' and 3' UTRs. However, it involves the SL2 of the 5' UTR not SL3, which disagrees with LinearTurboFold prediction and Ziv *et al.*. $\mathbf{B}$: RNAalifold (MFE) prediction for 25 genomes as listed in Fig. S9. RNAalifold did not find any 5'-3' pairs.

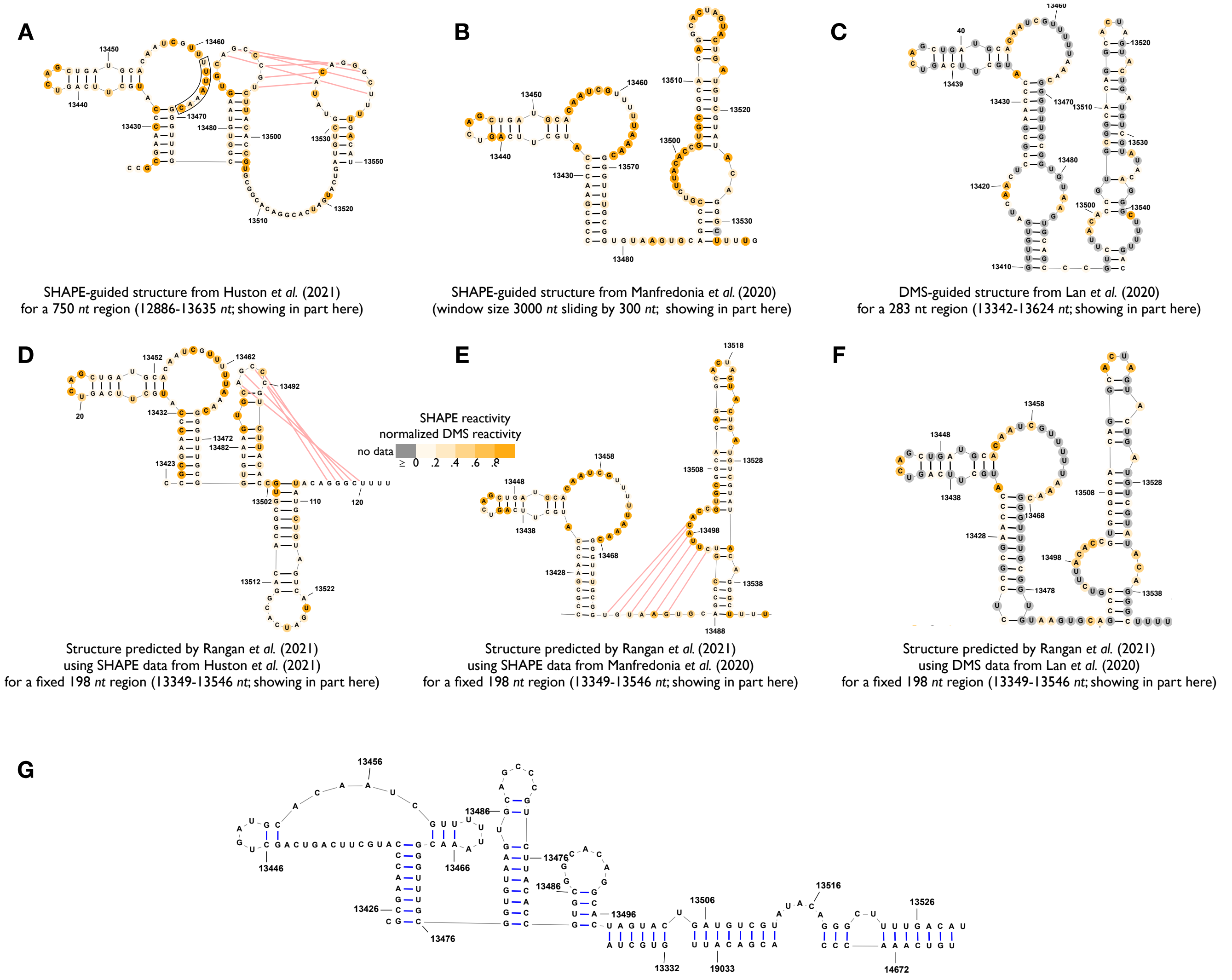

Fig. S11. Secondary structure predictions of SARS-CoV-2 for extended frameshifting stimulation element (13425-13545 nt). $\mathbf{A}$–$\mathbf{F}$: Experimentally-guided structures with different probing data for different regions. The structures in each column were estimated with the same experimental data but different regions. The structures in the second row were predicted by Rangan $et\ al.$ for a fixed region of 198 nt (18) . G: RNAalifold (MFE) prediction over 25 genomes as listed in Fig. S9.

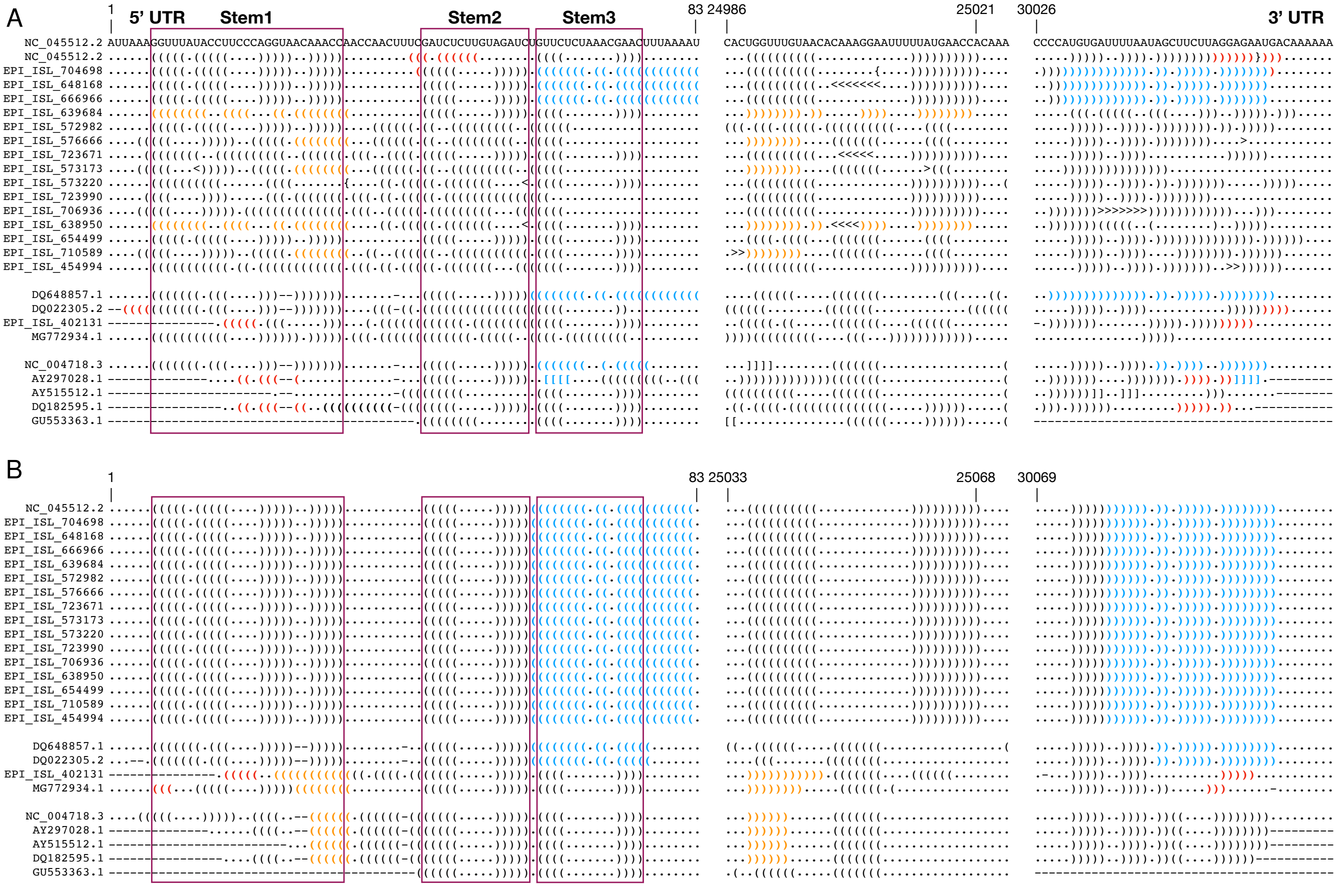

Fig. S12. ThreshKnot structures based on base-pairing probabilities from LinearPartition individually ($\mathbf{A}$) and LinearTurboFold jointly ($\mathbf{B}$), respectively, for 25 SARS-CoV-2 and SARS-related sequences as listed in Fig. S9. And these structures are aligned by the multiple sequence alignment from MAFFT ($\mathbf{A}$) and LinearTurboFold ($\mathbf{B}$), respectively. Three ranges (of $\sim$80 nt at the start of 5'UTR, of $\sim$50 nt at the end of 3' UTR and of $\sim$40 nt around 25000 nt) are concatenated. The inter-actions between the first and third ranges are blue if they are the same as interactions detected by Ziv $et\ al.$ (19), otherwise, they are red. And the base pairs between the first and second ranges are orange. LinearPartitioncan model the same end-to-end interactions detected by Ziv $et\ al.$ for 4 out of 16 SARS-CoV-2 (EPI_ISL_704698, EPI_ISL_648168, EPI_ISL_666966 and EPI_ISL_639684) and 4 out of 9 SARS-related (DQ648857.1, NC_004718.3, AY297028.1 and DQ022305.2) sequences. The probabilities of such end-to-end pairs are relatively small for EPI_ISL_639684 and DQ022305.2 (Fig. S13), therefore the pairs are not shown in the threshKnot structures. For other sequences, however, LinearPartition either cannot predict the end-to-end interactions or it predicts them in wrong locations (e.g., EPI_ISL_572982 and NC_045512.2). By contrast, LinearTurboFold propagates the correct structural information, i.e., base-pairing probabilities (Fig. S13), from these eight sequences to other homologs, and finally folds all the SARS-CoV-2 and two SARS-related sequences into the same structure. Meanwhile, LinearTurboFold predicts a long-range interaction involving the SL1 of 5' UTR and a segment around 25000 nt for other six SARS-related seqmuences. One SARS-relatedsequence (GU553363.1) can form neither interactions due to incomplete UTRs.

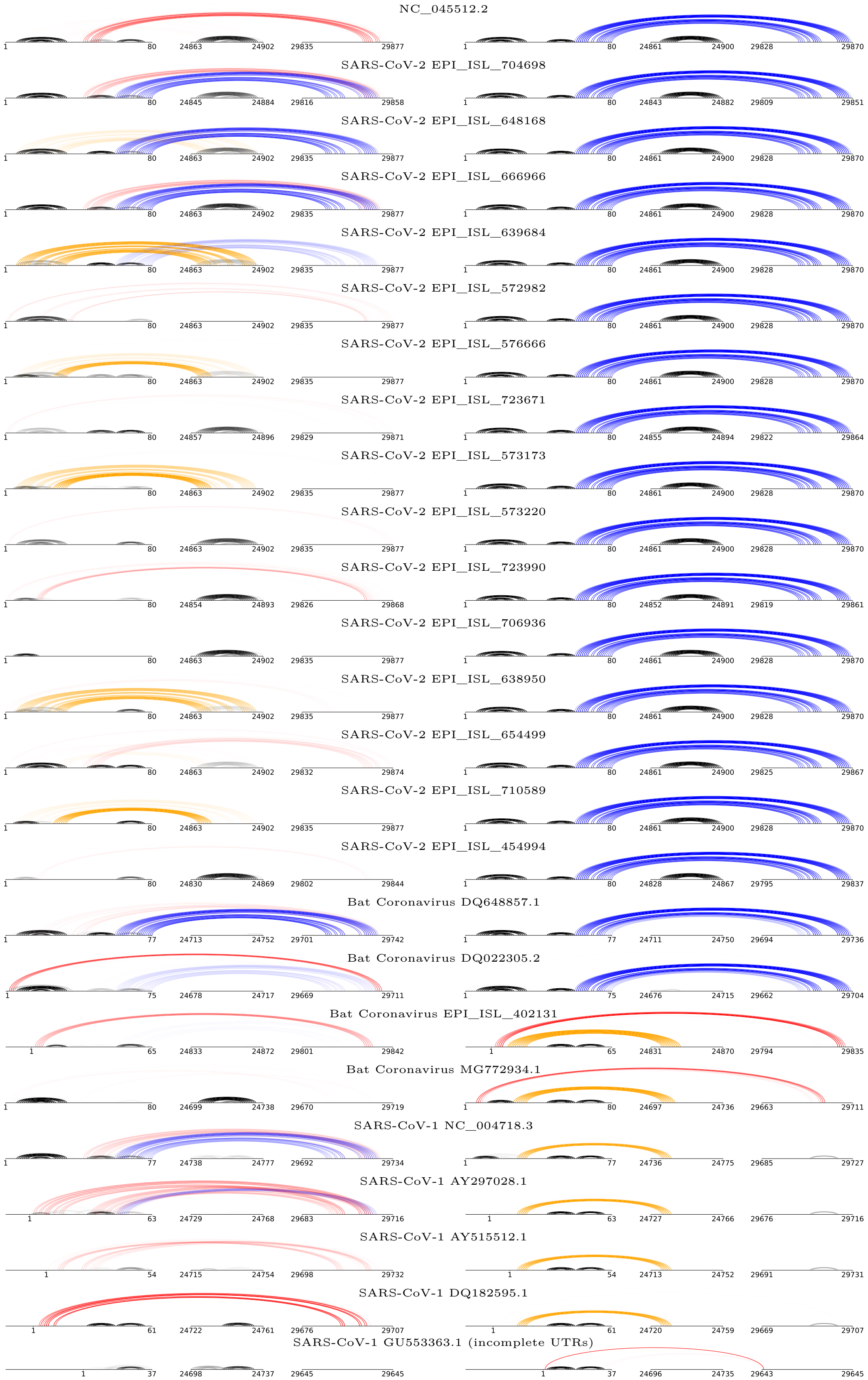

Fig. S13. Base-pairing probabilities predicted by LinearPartition individually (left column) and LinearTurboFold jointly (right column), respectively, for 25 SARS-CoV-2 and SARS-related genomes. The same three ranges as shown in Fig. S12 are concatenated. The arcs represent base pairs, whose transparency shows corresponding probabilities.

```
>NC_045512.2_Wuhan_seafood_market_pneumonia_virus_isolate_Wuhan-Hu-1__complete_genome:
CGUUCGGAUGCUCGAACUGCACCUCAUGGUCAUGUUAUGGUUGAGCUGGUAGCAGAACUCGAAGGCAUUCAGUACGGUCGUAGUGGUGAGACACUUGGUGUCCUUGUCCCUCAUGUGGGCGAAAUACCAGUGGCUUACCGC
(((.((((((((((.(((((((((...((((...)))).)))...))).))))...)))..))))))).).)))......(((((((.((((.(((((..(((.(((......)))))).))))))))).))))))))
>hCoV-19_Sweden_20-53189_2020_EPI_ISL_648168_2020-10-13_Europe:
CGUUCGGAUGCUCGAACUGCACCUCAUGGUCAUGUUAUGGUUGAGCUGGUAGCAGAACUCGAAGGCAUUCAGUACGGUCGUAGUGGUGAGACACUUGGUGUCCUUGUCCCUCAUGUGGGCGAAAUACCAGUGGCUUACCGC
(((.((((((((((.(((((((((...((((...)))).)))...))).))))...)))..))))))).).)))......(((((((.((((.(((((..(((.(((......)))))).))))))))).))))))))
>hCoV-19_Sweden_20-53470_2020_EPI_ISL_710589_2020-11-19_Europe:
CGUUCGGAUGCUCGAACUGCACCUCAUGGUCAUGUUAUGGUUGAGCUGGUAGCAGAACUCGAAGGCAUUCAGUACGGUCGUAGUGGUGAGACACUUGGUGUCCUUGUCCCUCAUGUGGGCGAAAUACCAGUGGCUUACCGC
(((.((((((((((.(((((((((...((((...)))).)))...))).))))...)))..))))))).).)))......(((((((.((((.(((((..(((.(((......)))))).))))))))).))))))))
>hCoV-19_England_MILK-B3D791_2020_EPI_ISL_704698_2020-11-01_Europe:
CGUUCGGAUGCUCGAACUGCACCUCAUGGUCAUGUUAUGGUUGAGCUGGUAGCAGAACUCGAAGGCAUUCAGUACGGUCGUAGUGGUGAGACACUUGGUGUCCUUGUCCCUCAUGUGGGCGAAAUACCAGUGGCUUACCGC
(((.((((((((((.(((((((((...((((...)))).)))...))).))))...)))..))))))).).)))......(((((((.((((.(((((..(((.(((......)))))).))))))))).))))))))
>hCoV-19_Scotland_QEUH-A77C42_2020_EPI_ISL_638950_2020-10-14_Europe:
CGUUCGGAUGCUCGAACUGCACCUCAUGGUCAUGUUAUGGUUGAGCUGGUAGCAGAACUCGAAGGCAUUCAGUACGGUCGUAGUGGUGAGACACUUGGUGUCCUUGUCCCUCAUGUGGGCGAAAUACCAGUGGCUUACCGC
(((.((((((((((.(((((((((...((((...)))).)))...))).))))...)))..))))))).).)))......(((((((.((((.(((((..((((.((......)))))).))))))))).))))))))
>hCoV-19_England_ALDP-9D8D3E_2020_EPI_ISL_576666_2020-09-18_Europe:
CGUUCGGAUGCUCGAACUGCACCUCAUGGUCAUGUUAUGGUUGAGCUGGUAGCAGAACUCGAAGGCAUUCAGUACGGUCGUAGUGGUGAGACACUUGGUGUCCUUGUCCCUCAUGUGGGCGAAAUACCAGUGGCUUACCGC
(((.((((((((((.(((((((((...((((...)))).)))...))).))))...)))..))))))).).)))......(((((((.((((.(((((..((((.((......)))))).))))))))).))))))))
>hCoV-19_England_ALDP-B5E5FA_2020_EPI_ISL_723671_2020-11-08_Europe:
CGUUCGGAUGCUCGAACUGCACCUCAUGGUCAUGUUAUGGUUGAGCUGGUAGCAGAACUCGAAGGCAUUCAGUACGGUCGUAGUGGUGAGACACUUGGUGUCCUUGUCCCUCAUGUGGGCGAAAUACCAGUGGCUUACCGC
(((.((((((((((.(((((((((...((((...)))).)))...))).))))...)))..))))))).).)))......(((((((.((((.(((((..(((((.(......)))))).))))))))).))))))))
>hCoV-19_Sweden_20-09856_2020_EPI_ISL_654499_2020-10-20_Europe:
CGUUCGGAUGCUCGAACUGCACCUCAUGGUCAUGUUAUGGUUGAGCUGGUAGCAGAACUCGAAGGCAUUCAGUACGGUCGUAGUGGUGAGACACUUGGUGUCCUUGUCCCUCAUGUGGGCGAAAUACCAGUGGCUUACCGC
(((.((((((((((.(((((((((...((((...)))).)))...))).))))...)))..))))))).).)))......(((((((.((((.(((((..(((.(((......)))))).))))))))).))))))))
>hCoV-19_England_QEUH-A61A50_2020_EPI_ISL_706936_2020-10-13_Europe:
CGUUCGGAUGCUCGAACUGCACCUCAUGGUCAUGUUAUGGUUGAGCUGGUAGCAGAACUCGAAGGCAUUCAGUACGGUCGUAGUGGUGAGACACUUGGUGUCCUUGUCCCUCAUGUGGGCGAAAUACCAGUGGCUUACCGC
(((.((((((((((.(((((((((...(((.....))).)))...))).))))...)))..))))))).).)))......(((((((.((((.(((((..(((.(((......)))))).))))))))).))))))))
>hCoV-19_England_CAMC-B7B1B7_2020_EPI_ISL_723990_2020-11-12_Europe:
CGUUCGGAUGCUCGAACUGCACCUCAUGGUCAUGUUAUGGUUGAGCUGGUAGCAGAACUCGAAGGCAUUCAGUACGGUCGUAGUGGUGAGACACUUGGUGUCCUUGUCCCUCAUGUGGGCGAAAUACCAGUGGCUUACCGC
(((.((((((((((.(((((((((...((((...)))).)))...))).))))...)))..))))))).).)))......(((((((.((((.(((((..(((.(((......)))))).))))))))).))))))))
>hCoV-19_England_CAMC-9C1B01_2020_EPI_ISL_573220_2020-09-09_Europe:
CGUUCGGAUGCUCGAACUGCACCUCAUGGUCAUGUUAUGGUUGAGCUGGUAGCAGAACUCGAAGGCAUUCAGUACGGUCGUAGUGGUGAGACACUUGGUGUCCUUGUCCCUCAUGUGGGCGAAAUACCAGUGGCUUACCGU
(((.((((((((((.(((((((((...((((...)))).)))...))).))))...)))..))))))).).))).......((((((.((((.(((((..(((.(((......)))))).))))))))).))))))).
>hCoV-19_Latvia_187_2020_EPI_ISL_639684_2020-10-03_Europe:
CGUUCGGAUGCUCGAACUGCACCUCAUGGUCAUGUUAUGGUUGAGCUGGUAGCAGAACUCGAAGGCAUUCAGUACGGUCGUAGUGGUGAGACACUUGGUGUCCUUGUCCCUCAUGUGGGCGAAAUACCAGUGGCUUACCGC
(((.((((((((((.(((((((((...((((...)))).)))...))).))))...)))..))))))).).)))......(((((((.((((.(((((..((((((......)))))).))))))))).))))))))
>hCoV-19_Wuhan_HB-WHCM-114_2020_EPI_ISL_454994_2020-03-02_Asia:
CGUUCGGAUGCUCGAACU---------------------------------GCAGAACUCGAAGGCAUUCAGUACGGUCGUAGUGGUGAGACACUUGGUGUCCUUGUCCCUCAUGUGGGCGAAAUACCAGUGGCUUACCGC
(((.((((((((((...---------------------------------.......)))..))))))).).)))......(((((((.((((.(((((..(((.(((......)))))).))))))))).))))))))
>hCoV-19_USA_CA-ALSR-4490_2020_EPI_ISL_666966_2020-10-30_NorUhAmerica:
CGUUCGGAUGCUCGAACUGCACCUCAUGGUCAUGUUAUGGUUGAGCUGGUAGCAGAACUCGAAGGCAUUCAGUACGGUCGUAGUGGUGAGACACUUGGUGUCCUUGUCCCUCAUGUGGGCGAAAUACCAGUGGCUUACCGC
(((.((((((((((.(((((((((...((((...)))).)))...))).))))...)))..))))))).).)))......(((((((.((((.(((((..((((((......)))))).))))))))).))))))))
>hCoV-19_England_ALDP-9B601F_2020_EPI_ISL_572982_2020-08-28_Europe:
CGUUCGGAUGCUCGAACUGCACCUCAUGGUCAUGUUAUGGUUGAGCUGGUAGCAGAACUCGAAGGCAUUCAGUACGGUCGUAGUGGUGAGACACUUGGUGUCCUUGUCCCUCAUGUGGGCGAAAUACCAGUGGCUUACCGC
(((.((((((((((.(((((((((...((((...)))).)))...))).))))...)))..))))))).).)))......(((((((.((((.(((((..(((.(((......)))))).))))))))).))))))))
>hCoV-19_England_CAMC-9C01A6_2020_EPI_ISL_573173_2020-09-08_Europe:
CGUUCGGAUGCUCGAACUGCACCUCAUGGUCAUGUUAUGGUUGAGCUGGUAGCAGAACUCGAAGGCAUUCAGUACGGUCGUAGUGGUGAGACACUUGGUGUCCUUGUCCCUCAUGUGGGCGAAAUACCAGUGGCUUACCGC
(((.((((((((((.(((((((((...((((...)))).)))...))).))))...)))..))))))).).)))......(((((((.((((.(((((..(((.(((......)))))).))))))))).))))))))
>NC_004718.3_SARS_coronavirus_Tor2__complete genome:
CGUUCUGAUGCCUUAAGCACCAAUCACGGCCACAAGGUCGUUGAGCUGGUUGCAGAAAUGGACGGCAUUCAGUACGGUCGUAGCGGUAUAACACUGGGAGUACUCGUGCCACAUGUGGGCGAAACCCCAAUUGCAUACCGC
(((.((((((((.(..((((((.((((((((....))))).)))..)))).))......)...))))).))).)))......((((((.....((((.((..((((.(((....))))))).))))))).....)))))))
>GU553363.1_SARS_coronavirus_HKU-39849_isolate_TCVSP-HARROD-00001__complete_genome:
CGUUCUGAUGCCUUAAGCACCAAUCACGGCCACAAGGUCGUUGAGCUGGUUGCAGAAAUGGACGGCAUUCAGUACGGUCGUAGCGGUAUAACACUGGGAGUACUCGUGCCACAUGUGGGCGAAACCCCAAUUGCAUACCGC
(((.((((((((.(..((((((.((((((((....))))).)))..)))).))......)...))))).))).)))......((((((.....((((.((..((((.(((....))))))).))))))).....)))))))
>AY515512.1_SARS_coronavirus_HC_SZ_61_03__complete_genome:
CGUUCUGAUGCCUUAAGCACCAAUCACGGCCACAAGGUCGUUGAGCUGGUUGCAGAAAUGGACGGCAUUCAGUACGGUCGUAGCGGUAUAACACUGGGAGUACUCGUGCCACAUGUGGGCGAAACCCCAAUUGCAUACCGC
(((.((((((((.((.((((((.((((((((....))))).)))..)))).)).....))...))))).))).)))......((((((.....((((.((..((((.(((....))))))).))))))).....)))))))
>AY297028.1_SARS_coronavirus_ZJ01__complete_genome:
CGUUCUGAUGCCUAAAGCACCUAUCACGGCCACAAGGUCGUUGAGCUGGUUGCAGAAAUGGACGGCAUUCAGUACGGUCGUAGCGGUAUAACACUGGGAGUACUCGUGCCACAUGUGGGCGAAACCCCAAUUGCAUACCGC
(((.((((((((....(((((..((((((((....))))).)))...))).)).........))))).))).)))......((((((.....((((.((..((((.(((....))))))).))))))).....)))))))
>DQ182595.1_SARS_coronavirus_ZJ0301_from_China__complete_genome:
CGUUCUGAUGCCUUAAGCACCAAUCACGGCCACAAGGUCGUUGAGCUGGUUGCAGAAAUGGACGGCAUUCAGUACGGUCGUAGCGGUAUAACACUGGGAGUACUCGUGCCACAUGUGGGCGAAACCCCAAUUGCAUACCGC
(((.((((((((.((.((((((.((((((((....))))).)))..)))).)).....))...))))).))).)))......((((((.....((((.((..((((.(((....))))))).))))))).....)))))))
>MG772934.1_Bat_SARS-like_coronavirus_isolate_bat-SL-CoVZXC21__complete_genome:
CGUUCUGAUGCCCGAACUGCACAUCAUGGCCAUGUUAUGGUCGAAUUAGUAGCAGAACUCGAUGGCAUUCAGUACGGUCGUAGUGGUGAGACACUUGGUGUUCUUGUCCCUCAUGUGGGAGAGGUACCAGUUGCUUACCGU
(((.((((((((((.((((((....((((((...)))))).....)).))))...)))..))))).))).))).......(((((((..(((.((((..(((.((((.....))).))))))))))..))))))).
>DQ022305.2_Bat_SARS_coronavirus_HKU3-1__complete_genome:
CGAUCUGAUGCUCUAAGCACCAAUCACGGCCACAAGGUUGUGGAAUUGGUUGCAGAAUUAGAUGGCAUUCAGUUCGGUCGUAGCGGUAUAACACUGGGAGUUCUCGUGCCACAUGUGGGCGAAACCCCAAUCGCAUACCGU
(((.((((((((((((((((((((((((((....)))))))..)))))).))....)))).))))).))).)))......((((((.....((((.(((.((((.(((....))))))))))))))).....)))))))
>DQ648857.1_Bat_coronavirus_(BtCoV_279_2005)__complete_genome:
CGUUCUGAUGCCCAGGGCACCAAUCACGGCUAUAAGGUCGUUGAAUUGGUUGCAGAAUUGGACGGCAUUCAGUACGGUCGUAGCGGUACAACACUGGGAGUCCUCGUGCCACAUGUGGGCGAAACCCCAGUUGCGUACCGU
(((.((((((((((..((((((((((((((....)))))).)).))))).)).....))...))))).))).)))......((((((...((((((.((..((((.(((....))))))).))))))))...)))))))
>BetaCoV_bat_Yunnan_RatG13_2013_EPI_ISL_402131:
CGUUCUGAUGCUCGAACUGCACCUCAUGGCCAUGUUAUGGUUGAGCUGGUAGCAGAACUUAAUGGCAUUCAGUAUGGUCGUAGUGGUGAGACACUCGGUGUCCUUGUCCCUUAUGUGGGCGAAACACCAGUGGUUUACCGC
(((.((((((((.((.(((((((((..((((((...))))))))...))).))))...))...))))).))).)))......(((((((.((((.(((((..(((((((....).)))))).))))))))).))))))))
```

Fig. S14. A glimpse of the whole MSA and aligned predicted structures for 25 genomes from LinearTurboFold, which are available in https://github.com/LinearFold/LinearTurboFold/blob/main/sars-cov-2_results/. Each genome corresponds to three lines: name, aligned sequence and aligned structure, respectively.

Table S1. Structure prediction and multiple sequence alignment accuracies. LinearTurboFold (1) and (2) use the same beam size (100) in LinearPartition, and different beam sizes of 100 and ∞ (i.e., exact search) in HMM alignment, respectively.

Structure Prediction Accuracy

	LinearTurboFold (1)	(2)	TurboFold II	LocARNA	MXSCARNA	LinearPartition	Vienna RNAfold	RNAalifold
					PPV			
SRP	0.801	0.801	**0.819**	0.698	0.485	0.662	0.673	0.629
telomerase	0.650	0.649	**0.685**	0.516	0.465	0.409	0.430	0.602
RNase P RNA	0.734	0.731	**0.752**	0.606	0.573	0.543	0.571	0.698
16S rRNA	0.615	0.610	0.608	0.586	**0.662**	0.467	0.464	0.628
overall	0.700	0.698	**0.716**	0.602	0.546	0.520	0.534	0.639
					Sensitivity			
SRP	**0.806**	0.806	0.743	0.693	0.488	0.700	0.682	0.218
telomerase	**0.832**	0.833	0.826	0.637	0.558	0.584	0.576	0.482
RNase P RNA	**0.828**	0.830	0.758	0.630	0.584	0.650	0.636	0.478
16S rRNA	0.620	0.623	0.584	0.622	**0.663**	0.511	0.469	0.605
overall	**0.772**	0.773	0.728	0.645	0.573	0.611	0.591	0.446
					F1 scores			
SRP	**0.804**	0.804	0.779	0.695	0.486	0.681	0.677	0.323
telomerase	0.730	0.730	**0.749**	0.570	0.507	0.481	0.492	0.535
RNase P RNA	**0.778**	0.777	0.755	0.618	0.578	0.592	0.602	0.567
16S rRNA	0.617	0.617	0.596	0.603	**0.662**	0.488	0.466	0.616
overall	**0.734**	0.734	0.722	0.623	0.559	0.562	0.561	0.525

Multiple Sequence Alignment Accuracy

	LinearTurboFold (1)	(2)	TurboFold II	LocARNA	MXSCARNA	LinearAlignment	MAFFT	RNAforester (7)
					PPV			
SRP	**0.463**	0.463	0.458	0.305	0.387	0.414	0.393	0.263
telomerase	**0.617**	0.616	0.615	0.311	0.554	0.575	0.572	0.239
RNase P RNA	**0.788**	0.789	0.787	0.615	0.692	0.744	0.759	0.258
16S rRNA	0.971	0.977	**0.977**	0.647	0.971	0.947	0.974	0.239
overall	**0.710**	0.711	0.709	0.470	0.651	0.670	0.675	0.250
					Sensitivity			
SRP	**0.443**	0.443	0.438	0.452	0.384	0.396	0.382	0.271
telomerase	**0.573**	0.573	0.572	0.470	0.523	0.540	0.529	0.262
RNase P RNA	**0.765**	0.766	**0.765**	0.596	0.684	0.724	0.738	0.286
16S rRNA	0.971	0.977	**0.977**	0.974	0.971	0.951	0.973	0.298
overall	**0.688**	0.690	**0.688**	0.623	0.641	0.653	0.656	0.280
					F1 scores			
SRP	**0.453**	0.452	0.448	0.364	0.385	0.405	0.388	0.267
telomerase	**0.594**	0.594	0.593	0.375	0.538	0.557	0.550	0.250
RNase P RNA	**0.776**	0.777	**0.776**	0.605	0.688	0.734	0.748	0.271
16S rRNA	0.971	0.977	**0.977**	0.778	0.971	0.949	0.973	0.265
overall	**0.699**	0.700	0.698	0.535	0.646	0.661	0.665	0.264

Table S2. Fully conserved base pairs across 25 complete SARS-CoV-2 and SARS-related genomes with compensatory mutations. The positions and nucleotide type of base pairs correspond to the reference sequence of SARS-CoV-2 (NC_0405512.2). The mutations are from the other 24 genomes.

5' End	3' End	Base Pair	Average Probability	Mutations	5' End	3' End	Base Pair	Average Probability	Mutations
90	121	GC	0.971	AU/GU	12931	12964	UA	0.851	AU/GC
97	115	AU	0.969	GU/GC	13069	13108	UA	1.000	CG
153	291	UA	0.972	CG	13078	13099	UA	1.000	UG/CG
159	282	GC	0.961	AU/GU	13216	13222	UA	0.958	UG/CG
189	217	GC	0.972	AU	13599	13628	UA	1.000	CG
358	385	UA	0.960	UG/CG	13638	13695	UA	0.986	AU
367	373	CG	0.961	UA	13641	13692	UA	0.977	CG
407	478	GC	0.936	AU	14091	14107	UA	0.989	CG
442	448	CG	0.960	UA/UG	14161	14194	UA	1.000	UG/CG
484	555	UA	0.877	AU	14205	14211	AU	0.933	CG
570	616	AU	0.946	UA/CG	14224	14251	AU	0.996	GU/GC
652	724	AU	0.947	GC	14355	14361	AU	0.996	GC
677	703	GC	0.933	AU	14487	14532	AU	0.973	GU/UA/UG/CG
880	889	AU	0.962	CG	14595	14604	UA	0.999	UG/CG
970	981	GC	0.963	AU	15435	15453	AU	0.778	GC
1231	1251	GC	0.968	AU	15582	15607	AU	0.993	GC
1949	1956	UA	0.929	UG/CG	16023	16032	UA	0.998	AU
2278	2303	UA	0.970	CG	16080	16110	CG	0.971	UA
2855	2875	CG	0.962	UA/UG	16089	16101	GC	1.000	AU
2896	2923	UA	0.973	AU/GU	16125	16155	AU	0.999	UA
2959	2986	UA	0.973	UG/CG	16230	16236	CG	0.999	UA
3712	3721	AU	0.977	GC	16677	16716	GC	1.000	AU
3913	3928	UA	0.979	AU/UG	17241	17256	UA	0.980	CG
3915	3926	AU	0.965	GC	17244	17253	AU	0.980	GC
4096	4108	UA	0.926	UG/CG	17304	17331	CG	0.981	UA
4189	4225	CG	0.980	GC/UG	18006	18054	UA	0.980	AU/GU
4603	4624	UA	0.980	UG/CG	18439	18468	UA	0.980	AU
4978	4987	UA	0.982	CG	18549	18561	AU	0.982	CG
5164	5203	GC	0.975	AU/GU	18717	18774	UA	0.983	UG/CG
5347	5374	UG	0.984	AU/GC/UA	19074	19098	UA	0.882	AU
5356	5371	UA	0.953	AU/GC	19386	19419	CG	0.986	UA/UG
5417	5428	UA	0.982	AU	19395	19410	UA	0.981	CG
5476	5521	AU	0.940	GU/GC	19707	19732	CG	0.981	UA
5482	5515	CG	0.984	UA/UG	19708	19731	AU	0.985	GU/GC
5739	5770	GC	0.984	AU	19917	19953	UA	0.980	AU
6034	6055	AU	0.983	GC	19929	19941	UA	0.984	AU
6037	6052	CG	0.982	UA	20172	20187	UA	0.977	CG
6154	6202	AU	0.987	GC	20217	20265	UA	0.939	CG
6328	6343	AU	0.988	UA	20223	20260	AU	0.981	GC
6364	6388	GC	0.989	AU	20523	20541	UA	0.988	CG
6367	6385	GC	0.989	AU	20841	20901	AU	0.988	GU/GC
6458	6490	AU	0.988	GC	20985	20997	AU	0.757	UA
6460	6488	UA	0.988	CG	21163	21201	AU	0.980	GU/GC
6903	6922	CG	0.895	UA	21300	21321	AU	0.988	GU/GC
6977	7006	GC	0.970	AU/GU	21411	21423	CG	0.989	UA
7103	7135	AU	0.891	GU/GC	21513	21523	CG	0.988	UA
7480	7531	UA	0.942	UG/CG	22837	22903	AU	0.988	GU/GC
7558	7597	AU	0.956	GC	23531	23548	AU	0.717	GC
7864	7876	AU	0.972	GU/GC	23621	23647	GC	0.991	AU
8146	8219	CG	0.993	UA	23797	23806	UA	0.931	UG/CG
8147	8218	AU	0.992	GU/GC	23980	24088	AU	0.978	GU/GC

Table S2 continued from previous page

5' End	3' End	Base Pair	Average Probability	Mutations	5' End	3' End	Base Pair	Average Probability	Mutations
8153	8212	UA	0.987	CG	23983	24085	AU	0.974	UA/CG
8317	8332	AU	0.995	GU/GC	24121	24152	AU	0.994	GC
8437	8458	AU	0.915	GU/GC	24553	24586	CG	0.996	UA
8698	8738	UA	0.824	CG	24757	24766	GC	0.974	GU/CG
8860	8881	CG	0.996	AU/UA	25336	25370	AU	0.906	GU/GC
9046	9079	UA	0.995	UG/CG	25991	26004	GC	0.996	AU/GU
9055	9070	AU	0.969	GC	26145	26190	UA	0.997	CG
9427	9433	UA	0.991	CG	26262	26305	GC	0.911	AU
9472	9511	AU	0.996	UA	26630	26658	AU	0.903	GC
9689	9703	AU	0.932	GC	26676	26706	AU	0.986	GC
9842	9874	UA	0.990	UG/CG	26939	26975	AU	0.996	CG
10213	10248	UA	0.997	CG	27412	27456	UA	0.998	UG/CG
10651	10669	UA	0.998	CG	27415	27453	GC	0.994	AU
10864	10906	AU	0.926	GC	27603	27613	CG	0.996	UA
10873	10898	AU	0.946	GC	27699	27744	UA	0.935	CG
10984	11026	AU	0.976	GC	27717	27725	GC	0.990	AU/GU
11782	11803	AU	0.981	GU/GC	28642	28664	UA	0.997	UG/CG
11788	11797	CG	1.000	UA	28910	28930	AU	0.997	GU/GC
11971	12013	AU	0.966	GU/GC	29567	29597	AU	0.999	GC
11989	11995	UA	0.763	UG/CG	29635	29651	CG	0.999	AU
12538	12577	UA	0.940	UG/CG	29637	29649	UA	0.991	CG

Table S3. Fully conserved structures among 25 genomes. Regions with compensatory mutations are annotated with alternative base pairs. Novel regions compared with Rangan *et al.* (20, 21) are annotated with stars.

Region	Sequence & Structure	Compensatory Mutations
45-59	GAUCUCUUGUAGAUC<br>(((((.....)))))	
84-127	CUGUGUGGCUGUCACUCGGCUGCAUGCUUAGUGCACUCACGCAG<br>((((((((.((.((((.(((.....))).)))))).))))))))	GC ->AU<br>AU ->GC
626-643*	GUUCUUCUUCGUAAGAAC<br>((((((......))))))	
1324-1341	UGCCACUACUUGUGGUUA<br>((((((.....))))).)	
1685-1698	GCCAUUAUUUUGGC<br>((((......))))	
1785-1800*	GUAAUUUUAAAGUUAC<br>((((((....))))))	
2818-2837*	AGUACUUAAUGAGAAGUGCU<br>(((((((......)))))))	
4973-4993	GUGUUUACAACAGUAGACAAC<br>((((((((....))))))).)	UA ->CG
5021-5033	AUGUCAAUGACAU<br>(((((...)))))	
6450-6498	UUGAGUGUAAUGUGAAAACUACCGAAGUUGUAGGAGACAUUAUACUUAA<br>((((((((((((.....((((.......))))...))))))))))))	AU ->GC<br>UA ->CG
8078-8084	CCAAUGG<br>((...))	
10494-10509*	GUGUUGGUUUUAACAU<br>((((((....))))))	
11131-11140	UGCUUUUGCA<br>(((....)))	
12203-12218*	UUGAAGAAGUCUUUGA<br>((((((....))))))	
12257-12268	CAACGUAAGUUG<br>((((....))))	
12386-12412	GAUAAUGAUGCACUCAACAACAUUAUC<br>(((((((.((.......)).)))))))	
12672-12685	CUGUCAAAUUACAG<br>((((......))))	
12904-12926*	UAGGUUUGUUACAGACACACCUA<br>(((((.((((...)))).)))))	
12970-12988*	CAACCUAAAUAGAGGUAUG<br>(((((......)))).))	
13409-13422*	AGUUGUGAUCAACU<br>(((((....)))))	
14729-14769	AGGAAGGAAGUUCUGUUGAAUUAAAACACUUCUUCUUUGCU<br>((.(((((((...((((.......))))...))))))).))	
14773-14790	GAUGGUAAUGCUGCUAUC<br>(((((((....)))))))	
14794-14818	GAUUAUGACUACUAUCGUUAUAAUC<br>(((((((((.......)))))))))	
15254-15269*	UUUAUAGUGAUGUAGA<br>((((((....))))))	
15430-15458*	AGUGAAAUGGUCAUGUGUGGCGGUUCACU<br>((((((.(.((((....)))).)))))))	AU ->GC
15502-15509*	GCUUAUGC<br>((....))	
15618-15628	ACUUUAUGAGU<br>((((...))))	
15775-15801*	AUAAAGAACUUUAAGUCAGUUCUUUAU<br>((((((((((.......))))))))))	
16013-16042*	GGUUCGUGUCUUUAGCUAUAGAUGCUUACC<br>(((..((((((........))))))..)))	UA ->AU
16180-16194	AGGUAUUGGGAACCU<br>((((.......))))	
16955-16973*	UAGUGCCACAAGAGCACUA<br>(((((((....).))))))	

Table S3 continued from previous page

Region	Sequence & Structure	Compensatory Mutations
17236-17262	AUACCUGCACGUGCUCGUGUAGAGUGU<br>(((((((((((....))))))).))))	UA ->CG<br>AU ->GC
17462-17470	GCACAUUGC<br>(((...)))	
18547-18563*	UUAAGUGACACACUUAA<br>(((((((...)))))))	AU ->CG
18660-18674*	UUGUCUAUGUGAUAG<br>(((((.....)))))	
18848-18872*	CUAGUUGUGAUGCAAUCAUGACUAG<br>(((((((((((...)))))))))))	
19036-19043*	GGUAACCC<br>((....))	
19973-20002*	CACUCACUGUCUUUUUUGAUGGUAGAGUUG<br>(((((((((((......)))))).)))).)	
20103-20127*	UCUUAAUGGAGUCACAUUAAUUGGA<br>((((((((......)))))...)))	
20709-20719*	GCAAAGAAUGC<br>(((.....)))	
23101-23110*	UUCUUUUGAA<br>(((....)))	
23119-23128	UGCACCAGCA<br>(((....)))	
23796-23807	AUUCAACUGAAU<br>((((....))))	UA ->CG
24305-24319	GUUCUCUAUGAGAAC<br>((((((...))))))	
25380-25407*	CAUAAACGAACUUAUGGAUUUGUUUAUG<br>((((((((((((...)).))))))))))	
25937-25959	AUGACUACCAGAUUGGUGGUUAU<br>((((((((((...))))))))))	
26209-26224	GUGCCUUUGUAAGCAC<br>((((........))))	
26358-26379	AUUGUGUGCGUACUGCUGCAAU<br>((((((.(((...)))))))))	
26581-26600	GGAACCUAGUAAUAGGUUUC<br>((((((((....))))))))	
26713-26740	GUUUUGUGCUUGCUGCUGUUUACAGAAU<br>((((((((...((....)).))))))))	
27635-27651*	CAGUUUCACCUAAACUG<br>((((((.....))))))	
28755-28766	UUCCUCAAGGAA<br>((((....))))	
29145-29166	UAAUCAGACAAGGAACUGAUUA<br>(((((((........)))))))	
29210-29225	GCGUUCUUCGGAAUGU<br>((((((....))))))	
29240-29253	GAAGUCACACCUUC<br>((((......))))	
29288-29306	UUGGAUGACAAAGAUCCAA<br>((((((.......))))))	
29321-29347	GUCAUUUUGCUGAAUAAGCAUAUUGAC<br>((((...((((.....))))...))))	
29470-29488*	UUUGGAUGAUUUCUCCAAA<br>((((((.(....)))))))	
29548-29613	CACAAGGCAGAUGGGCUAUAUAAACGUUUUCGCUUUUCCGUUUACGAUAUAUAGUCUACUCUUGUG<br>((((((..((.(((((((((((..(((...((......))...))).)))))))))))))))))))	UA ->CG

Table S4. Accessibility and conservation of target regions for public RT-PCR forward/reverse primers and probes (22). The accessibility is computed by LinearTurboFold, and it is underlined if larger than zero. The conservation on 9 SARS-related genomes is the number of mutated sites. The conservation on the ∼2M SARS-CoV-2 dataset is the percentage of exact matches, which is underlined or bold if less than 0.97 or 0.5, respectively. (The average sequence identity of SARS-CoV-2 genomes is 0.9987, and the average length of primers and probes is 23 nt. Therefore, the probability of randomly sampling a region of length 23 nt without mutations is $0.9987^{23} \approx 0.97$).

Gene	Institute	Forward Primer / Probe / Reverse Primer			Conservation	
		Start	Length	Accessibility	SARS-related # mut. site	SARS-CoV-2 (2M) exact match
ORF1ab nsp9	Institut Pasteur (1)	12689 / 12717 / 12779	18 / 21 / 18	0.0000 / 0.0000 / 0.0160	4 / 3 / 5	0.9989 / 0.9967 / 0.9829
ORF1ab nsp10	China CDC (1)	13341 / 13377 / 13441	21 / 30 / 19	0.0000 / 0.0000 / 0.0000	4 / 3 / 11	0.9937 / 0.9875 / 0.9868
ORF1ab nsp12 (RdRp)	Institut Pasteur (2)	14079 / 14105 / 14166	19 / 19 / 20	0.0000 / 0.0001 / 0.0000	4 / 8 / 9	0.9978 / 0.9332 / 0.9941
	Charite Germany (1)	15430 / 15469 / 15504	22 / 25 / 26	0.0000 / 0.0000 / 0.0000	0 / 6 / 1	0.9167 / 0.9938 / 0.9982
ORF1ab nsp14	HKU (1)	18777 / 18849 / 18888	20 / 24 / 21	0.0000 / 0.0000 / 0.0000	1 / 1 / 3	0.9958 / 0.9969 / 0.9933
E	Charite Germany (2)	26268 / 26332 / 26359	26 / 26 / 22	0.0000 / 0.0000 / 0.0000	0 / 0 / 0	0.9958 / 0.9969 / 0.9933
N	CDC (1)	28286 / 28309 / 28334	20 / 24 / 24	0.0000 / 0.0000 / 0.0000	12 / 2 / 8	0.9913 / 0.9762 / 0.9934
	NIH Thailand	28319 / 28341 / 28357	20 / 16 / 19	0.0000 / 0.0026 / 0.0000	3 / 6 / 6	0.9908 / 0.9953 / 0.9927
	CDC (2)	28680 / 28704 / 28731	22 / 24 / 21	0.0000 / 0.0000 / 0.0010	4 / 4 / 2	0.9862 / 0.9796 / 0.9895
	Charite Germany (3)	28705 / 28753 / 28813	19 / 25 / 20	0.0000 / 0.0003 / 0.0000	2 / 0 / 6	0.9914 / 0.9920 / 0.9858
	China CDC (2)	28880 / 28934 / 28957	22 / 20 / 22	0.0710 / 0.0000 / 0.0000	5 / 7 / 4	**0.2734** / 0.9911 / **0.4844**
	NIID Japan	29124 / 29222 / 29262	20 / 20 / 20	0.0000 / 0.0000 / 0.0000	6 / 1 / 7	0.9953 / 0.9785 / 0.9853
	HKU (2)	29144 / 29179 / 29235	22 / 20 / 19	0.0000 / 0.0000 / 0.0000	2 / 2 / 1	0.9904 / 0.9945 / 0.9895
	CDC (3)	29163 / 29188 / 29212	20 / 23 / 18	0.0000 / 0.0000 / 0.0000	2 / 6 / 2	0.9892 / 0.9797 / 0.9901

Table S5. Accessible and conserved regions with two kinds of constraints on conservation: 1) at most three mutations on SARS-related genomes; 2) the average sequence identity on the SARS-CoV-2 dataset at least 0.999. The start positions and sequences correspond to the reference sequence of SARS-CoV-2 (NC_0405512.2). The accessibilities are calculated from folding with homologs (LinearTurboFold) and single sequence folding (LinearPartition), respectively. We searched for these regions among human representative transcript set (RefSeq Select RNA sequences, refseq_select) using BLAST, and several regions have the exact matches with human transcripts (underlined). Using single sequence folding can only get one accessible region (bold). The conservation of these regions on 9 SARS-related genomes is the number of mutated sites. The table also shows two types of conservations on a large SARS-CoV-2 dataset containing $\sim$2M genomes submitted to GISAID up to June 30, 2021: the average sequence identity with reference sequence, and the percentage of exact matches of the whole region, respectively.

Region	Start	Length	Sequence	Gene	Accessibility				Conservation		BLAST Match	GC (%)
					LinearTurboFold (Homologous Folding)			Single Seq.	SARSr (9)	SARS-CoV-2 (2M)		
					Average	Range	ΔG(kcal/mol)	Folding Range	# Mut. Sites	Identity / Exact		
Region 1	739	18	AGAAAACUGGAACACUAA	ORF1ab nsp1	0.71 $\pm$ 0.04	0.62 – 0.76	0.22 $\pm$ 0.04	0.00 – 0.00	2/18	0.9998 / 0.9970	14/18	33
Region 2	995	17	CGUUCUGAAAAGAGCUA		0.99 $\pm$ 0.00	0.99 – 0.99	0.01 $\pm$ 0.00	0.00 – 0.00	3/17	0.9999 / 0.9985	14/17	41
Region 3	998	17	UCUGAAAAGAGCUAUGA	ORF1ab nsp2	1.00 $\pm$ 0.00	1.00 – 1.00	0.00 $\pm$ 0.00	0.00 – 0.00	3/17	0.9999 / 0.9984	15/17	35
Region 4	1001	15	GAAAAGAGCUAUGAA		0.74 $\pm$ 0.08	0.51 – 0.80	0.19 $\pm$ 0.07	0.00 – 0.00	3/15	0.9999 / 0.9985	15/15	33
Region 5	6765	16	AUUAUAUGCCUUAUUU		0.96 $\pm$ 0.01	0.95 – 0.97	0.02 $\pm$ 0.00	0.30 – 0.40	3/16	1.0000 / 0.9993	14/16	19
Region 6	6767	15	UAUAUGCCUUAUUUC	ORF1ab nsp3 (PLpro)	0.96 $\pm$ 0.01	0.95 – 0.97	0.02 $\pm$ 0.00	0.37 – 0.42	3/15	0.9999 / 0.9981	15/15	27
Region 7	7691	22	CAGUUUAAAAGACCAAUAAAUC		0.77 $\pm$ 0.03	0.69 – 0.83	0.16 $\pm$ 0.03	0.00 – 0.00	3/22	1.0000 / 0.9991	20/22	27
Region 8	9527	18	UCAUUCACUGUACUCUGU		0.66 $\pm$ 0.03	0.60 – 0.70	0.26 $\pm$ 0.03	0.00 – 0.00	3/18	0.9997 / 0.9945	15/18	39
Region 9	9530	17	UUCACUGUACUCUGUUU		0.64 $\pm$ 0.03	0.57 – 0.68	0.28 $\pm$ 0.03	0.00 – 0.00	3/17	0.9998 / 0.9965	15/17	35
Region 10	9905	15	UACAAGUAUUUUAGU	ORF1ab nsp4	0.75 $\pm$ 0.10	0.51 – 0.82	0.18 $\pm$ 0.10	0.00 – 0.00	2/15	0.9999 / 0.9980	15/15	20
Region 11	10010	17	CUUUACCAACCACCACA		0.75 $\pm$ 0.06	0.54 – 0.79	0.18 $\pm$ 0.06	0.00 – 0.01	3/17	0.9999 / 0.9989	15/17	47
Region 12	11536	25	UAUUGUUUUUAUGUGUGUUGAGUAU		0.67 $\pm$ 0.06	0.59 – 0.78	0.25 $\pm$ 0.05	0.00 – 0.01	3/25	0.9998 / 0.9961	17/25	24
Region 13	11540	22	GUUUUUAUGUGUGUUGAGUAUU	ORF1ab nsp6	0.79 $\pm$ 0.08	0.69 – 0.92	0.15 $\pm$ 0.06	0.00 – 0.02	3/22	0.9998 / 0.9965	17/22	27
Region 14	11543	20	UUUAUGUGUGUUGAGUAUUG		0.78 $\pm$ 0.08	0.69 – 0.92	0.15 $\pm$ 0.06	0.00 – 0.00	3/20	0.9998 / 0.9970	17/20	30
Region 15	11547	19	UGUGUGUUGAGUAUUGCCC		0.79 $\pm$ 0.08	0.69 – 0.92	0.15 $\pm$ 0.06	0.00 – 0.00	3/19	0.9998 / 0.9954	15/19	47
Region 16	13454	15	CAAUCGUUUUUAAAC	ORF1ab nsp11	0.96 $\pm$ 0.04	0.88 – 0.98	0.02 $\pm$ 0.02	0.07 – 0.11	3/15	0.9998 / 0.9972	14/15	27
Region 17	15141	22	CAAUAGACAGUUUCAUCAAAAA		0.61 $\pm$ 0.05	0.50 – 0.67	0.30 $\pm$ 0.06	0.00 – 0.00	3/22	0.9999 / 0.9986	18/22	27
Region 18	15890	15	CAAUGCUAGUUAAAC	ORF1ab nsp12 (RdRp)	0.63 $\pm$ 0.06	0.50 – 0.68	0.29 $\pm$ 0.06	0.00 – 0.23	1/15	0.9999 / 0.9991	13/15	33
Region 19	15997	16	ACACUUAUGAUUGAAC		0.72 $\pm$ 0.03	0.65 – 0.76	0.20 $\pm$ 0.03	0.00 – 0.40	2/16	1.0000 / 0.9997	13/16	31
Region 20	17194	22	AAGGCAUUAAAAUAUUUGCCUA		1.00 $\pm$ 0.00	0.99 – 1.00	0.00 $\pm$ 0.00	0.00 – 0.00	3/22	0.9999 / 0.9989	16/22	27
Region 21	18032	17	CUUUACAAGCUGAAAAU	ORF1ab nsp13 (helicase)	0.67 $\pm$ 0.05	0.57 – 0.73	0.25 $\pm$ 0.05	0.00 – 0.00	2/17	0.9999 / 0.9978	14/17	29
Region 22	18035	15	UACAAGCUGAAAAUG		0.91 $\pm$ 0.10	0.54 – 0.95	0.07 $\pm$ 0.08	0.00 – 0.02	1/15	1.0000 / 0.9993	13/15	33
Region 23	18036	17	ACAAGCUGAAAAUGUAA		0.93 $\pm$ 0.09	0.57 – 0.97	0.05 $\pm$ 0.08	0.00 – 0.02	2/17	0.9998 / 0.9992	15/17	29
Region 24	20134	20	GUAAAAACACAGUUCAAUUA	ORF1ab nsp15	0.62 $\pm$ 0.05	0.52 – 0.68	0.29 $\pm$ 0.05	0.00 – 0.07	3/20	0.9998 / 0.9959	14/20	25
Region 25	20135	21	UAAAAACACAGUUCAAUUAUU		0.63 $\pm$ 0.04	0.54 – 0.68	0.29 $\pm$ 0.04	0.00 – 0.07	3/21	0.9998 / 0.9967	14/21	19
Region 26	25546	17	CUUCUUGCUGUUUUUCA	ORF3a	0.94 $\pm$ 0.02	0.89 – 0.95	0.04 $\pm$ 0.01	0.00 – 0.02	1/17	0.9998 / 0.9904	16/17	35
**Region 27**	**27132**	**15**	**UAUAAAUUAAACACA**	**M**	**0.70 $\pm$ 0.01**	**0.68 – 0.72**	**0.22 $\pm$ 0.01**	**0.65 – 0.79**	3/15	0.9998 / 0.9963	15/15	13
Region 28	27525	16	ACCAUUUCAUCCUCUA	ORF7a	0.99 $\pm$ 0.00	0.99 – 1.00	0.00 $\pm$ 0.00	0.00 – 0.05	3/16	0.9993 / 0.9927	14/16	38
Region 29	28402	16	AGGUUUACCCAAUAAU		1.00 $\pm$ 0.00	0.99 – 1.00	0.00 $\pm$ 0.00	0.00 – 0.81	1/16	0.9999 / 0.9985	14/16	31
Region 30	28690	15	GAAUACACCAAAAGA		0.78 $\pm$ 0.01	0.77 – 0.78	0.16 $\pm$ 0.00	0.01 – 0.52	3/33	0.9992 / 0.9879	14/15	33
Region 31	28691	20	AAUACACCAAAAGAUCACAU	N	0.77 $\pm$ 0.01	0.76 – 0.78	0.16 $\pm$ 0.00	0.00 – 0.52	3/20	0.9994 / 0.9883	15/20	30
Region 32	28694	18	ACACCAAAAGAUCACAUU		0.77 $\pm$ 0.01	0.76 – 0.78	0.16 $\pm$ 0.00	0.01 – 0.52	3/18	0.9994 / 0.9886	16/18	33
Region 33	29075	15	UACAAUGUAACACAA		1.00 $\pm$ 0.00	1.00 – 1.00	0.00 $\pm$ 0.00	0.01 – 0.83	3/15	0.9996 / 0.9942	12/15	27

Table S6. Accessible and conserved regions with a loose constraint on conservation: the average sequence identity on the ∼2M SARS-CoV-2 dataset is at least 0.999. The table keeps the same format as Tab. S5 and only displays new regions not included in that table.

Region	Start	Length	Sequence	Gene	Accessibility: LinearTurboFold (Homologous Folding) Average	Range	ΔG(kcal/mol)	Single Seq. Folding Range	Conservation: SARSr (9) # Mut. Sites	SARS-CoV-2 (2M) Identity / Exact	BLAST Match	GC (%)
Region 1	1094	19	UUAAAUUCCAUAAUCAAGA		0.86 ± 0.03	0.80 – 0.89	0.10 ± 0.02	0.01 – 0.10	8/19	0.9998 / 0.9963	17/19	21
Region 2	1301	19	ACUGAGAAUUUGACUAAAG		0.75 ± 0.05	0.64 – 0.79	0.18 ± 0.04	0.00 – 0.00	8/19	0.9997 / 0.9950	14/19	32
Region 3	1359	18	UUGUUAAAAUUUAUUGUC	ORF1ab nsp2	0.75 ± 0.02	0.72 – 0.81	0.17 ± 0.02	0.09 – 0.22	7/18	0.9999 / 0.9989	16/18	17
Region 4	1420	18	CGAAUACCAUAAUGAAUC		0.94 ± 0.00	0.94 – 0.95	0.04 ± 0.00	0.01 – 0.03	7/18	0.9997 / 0.9941	13/18	33
Region 5	2550	19	AUUUACAACCAUUAGAACA		0.93 ± 0.03	0.89 – 0.96	0.04 ± 0.02	0.24 – 0.31	12/19	0.9998 / 0.9971	13/19	26
Region 6	3648	15	UUCAACUUCUUAAGA		0.93 ± 0.02	0.91 – 0.96	0.04 ± 0.01	0.00 – 0.03	8/15	0.9999 / 0.9980	14/15	27
Region 7	3733	19	UGACCCUAUACAUUCUUUA		0.91 ± 0.01	0.89 – 0.91	0.06 ± 0.00	0.00 – 0.01	8/19	0.9996 / 0.9928	13/19	32
Region 8	4405	17	ACAUGCAGAAGAAACAC	ORF1ab nsp3	0.55 ± 0.02	0.51 – 0.59	0.36 ± 0.02	0.00 – 0.00	6/17	0.9999 / 0.9987	15/17	41
Region 9	4406	21	CAUGCAGAAGAAACACGCAAA	(PLpro)	0.75 ± 0.03	0.71 – 0.80	0.18 ± 0.02	0.00 – 0.00	7/21	0.9999 / 0.9975	17/21	43
Region 10	4864	26	AAGUGUAUAUUACACUAGUAAUCCUA		0.85 ± 0.07	0.60 – 0.88	0.11 ± 0.06	0.00 – 0.00	14/26	0.9999 / 0.9975	16/26	27
Region 11	5773	23	UAAACAUAUAACUUCUAAAGAAA		0.64 ± 0.04	0.57 – 0.70	0.28 ± 0.04	0.00 – 0.21	9/23	0.9998 / 0.9961	16/23	17
Region 12	6129	16	UUAAAGUUACAUUUUU		0.97 ± 0.04	0.79 – 0.98	0.02 ± 0.03	0.01 – 0.11	6/16	1.0000 / 0.9996	16/16	13
Region 13	6499	32	ACCAGCAAAUAAUAGUUUAAAAAUUACAGAAG		0.61 ± 0.02	0.55 – 0.63	0.31 ± 0.03	0.00 – 0.00	15/32	0.9997 / 0.9941	19/32	25
Region 14	6622	19	GAAAACCCUUGCUACUCAU		0.95 ± 0.00	0.94 – 0.96	0.03 ± 0.00	0.00 – 0.01	8/19	0.9993 / 0.9869	15/19	42
Region 15	6697	15	UUUUCUUAACAAAGU		0.96 ± 0.00	0.95 – 0.97	0.03 ± 0.00	0.00 – 0.00	11/15	0.9995 / 0.9920	14/15	20
Region 16	7010	15	GCUUUAGGUGUUUUA		0.76 ± 0.03	0.69 – 0.79	0.17 ± 0.02	0.00 – 0.01	7/15	0.9999 / 0.9982	14/15	33
Region 17	7073	20	UAUUUGAACUCUACUAAUGU		0.85 ± 0.02	0.83 – 0.88	0.10 ± 0.01	0.00 – 0.24	7/20	0.9998 / 0.9967	15/20	25
Region 18	7725	19	CUUCUUACAUCGUUGAUAG		0.76 ± 0.06	0.69 – 0.86	0.17 ± 0.05	0.00 – 0.00	8/19	0.9996 / 0.9930	15/19	37
Region 19	9336	15	UAAAUUUACUUACUA	ORF1ab nsp4	1.00 ± 0.00	1.00 – 1.00	0.00 ± 0.00	0.00 – 0.95	8/15	0.9998 / 0.9976	13/15	13
Region 20	9555	15	UUUACUCAUUCUUAC		0.89 ± 0.01	0.88 – 0.91	0.07 ± 0.01	0.00 – 0.62	9/15	0.9995 / 0.9927	13/15	27
Region 21	11629	18	UUUUUGUACUUGUUACUU	ORF1ab nsp6	0.77 ± 0.07	0.69 – 0.88	0.16 ± 0.05	0.00 – 0.27	8/18	0.9998 / 0.9981	14/18	22
Region 22	12825	17	AUUUACAGGAUUUGAAA		0.75 ± 0.06	0.62 – 0.82	0.18 ± 0.05	0.00 – 0.01	9/17	0.9999 / 0.9991	16/17	24
Region 23	14170	16	AUAUUAACCUUGACCA	ORF1ab nsp12 (RdRp)	0.90 ± 0.01	0.88 – 0.92	0.06 ± 0.01	0.00 – 0.27	7/16	0.9997 / 0.9948	14/16	31
Region 24	16339	18	AUAUCAACAUCACAUAAA	ORF1ab nsp13	0.96 ± 0.06	0.83 – 1.00	0.02 ± 0.04	0.00 – 0.13	5/18	0.9999 / 0.9988	15/18	22
Region 25	17651	16	UUAAAAUGUUUUAUAA	(helicase)	0.96 ± 0.00	0.95 – 0.97	0.02 ± 0.00	0.00 – 0.17	5/16	0.9999 / 0.9988	15/16	6
Region 26	20652	16	AUUACAAUCUAGUCAA	ORF1ab nsp15	0.73 ± 0.02	0.70 – 0.77	0.19 ± 0.02	0.00 – 0.38	6/16	0.9995 / 0.9918	13/16	25
Region 27	20844	24	CUAUAAUAUGAGAGUUAUACAUUU	ORF1ab nsp16	1.00 ± 0.00	1.00 – 1.00	0.00 ± 0.00	0.00 – 0.02	7/24	0.9999 / 0.9982	14/24	21
Region 28	21622	16	CAGAACUCAAUUACCC		0.81 ± 0.03	0.78 – 0.89	0.13 ± 0.02	0.00 – 0.36	15/16	0.9992 / 0.9872	13/16	44
Region 29	21922	16	UAAUAACGCUACUAAU		0.96 ± 0.01	0.93 – 0.97	0.03 ± 0.01	0.00 – 0.00	7/16	0.9999 / 0.9988	14/16	25
Region 30	21950	15	GUCUGUGAAUUUCAA		0.73 ± 0.05	0.67 – 0.82	0.19 ± 0.04	0.00 – 0.11	6/15	0.9999 / 0.9983	14/15	33
Region 31	22876	24	UAACAAUCUUGAUUCUAAGGUUGG		0.94 ± 0.04	0.86 – 0.97	0.04 ± 0.02	0.00 – 0.00	23/24	0.9993 / 0.9825	16/24	33
Region 32	23031	16	UUCCUUUACAAUCAUA		0.98 ± 0.00	0.98 – 0.98	0.01 ± 0.00	0.00 – 0.04	14/16	0.9995 / 0.9920	13/16	25
Region 33	24058	15	CUUCAUCAAACAAUA	S	0.75 ± 0.13	0.51 – 0.87	0.19 ± 0.12	0.00 – 0.00	5/15	0.9999 / 0.9988	14/15	27
Region 34	24166	19	AAUGAUUGCUCAAUACACU		0.73 ± 0.06	0.61 – 0.78	0.20 ± 0.05	0.00 – 0.16	8/19	0.9999 / 0.9977	17/19	32
Region 35	24170	16	AUUGCUCAAUACACUU		0.56 ± 0.03	0.50 – 0.60	0.36 ± 0.04	0.00 – 0.08	8/16	0.9999 / 0.9978	13/16	31
Region 36	24368	17	GACUCACUUUCUUCCAC		0.68 ± 0.07	0.53 – 0.74	0.24 ± 0.07	0.00 – 0.01	8/17	0.9992 / 0.9877	16/17	47
Region 37	25015	18	GUUAGAUAAAUAUUUUAA		0.78 ± 0.04	0.70 – 0.83	0.15 ± 0.03	0.00 – 0.03	6/18	0.9999 / 0.9987	14/18	11
Region 38	27312	16	UAAAAAUUUAUCUAAG	ORF6	0.76 ± 0.01	0.74 – 0.77	0.17 ± 0.01	0.00 – 0.19	7/16	0.9999 / 0.9977	14/16	13

Table S7. Accessible regions by single sequence folding (applying LinearSampling on the SARS-CoV-2 reference sequence alone). The accessibility of the corresponding regions in other 15 SARS-CoV-2 genomes are calculated for each sequence separately. Except for the region in the M gene (in bold), all accessible regions on the reference sequence are not accessible on the other sequences, and always result in a wide range of accessibilities. By contrast, LinearTurboFold is able to find regions that are accessible across all 16 SARS-CoV-2 genomes thanks to fact that consensus folding is determined across the homologous sequences (Tab. S5).

Start	Length	Sequence	Gene	Accessibility		
				Reference Sequence	SARS-CoV-2 sequences (15)	
					Average	Range
9555	15	UUUACUCAUUCUUAC	ORF1ab	0.61	0.52 ± 0.19	0.00 – 0.62
20147	17	UCAAUUAUUAUAAGAAA	ORF1ab	0.56	0.07 ± 0.16	0.00 – 0.55
23705	16	CCCACAAAUUUUACUA	S	0.71	0.55 ± 0.31	0.01 – 0.90
23985	15	AUCCAUCAAAACCAA	S	0.62	0.59 ± 0.15	0.05 – 0.72
25700	20	CCCCUUUUCUCUAUCUUUAU	S	0.97	0.17 ± 0.26	0.00 – 0.98
**27129**	**18**	**AACUAUAAAUUAAACACA**	**M**	**0.77**	**0.76 ± 0.04**	**0.62 – 0.78**
28433	15	ACCGCUCUCACUCAA	N	0.55	0.27 ± 0.27	0.00 – 0.70
28691	17	AAUACACCAAAAGAUCA	N	0.54	0.45 ± 0.22	0.01 – 0.67
29074	16	AUACAAUGUAACACAA	N	0.83	0.56 ± 0.40	0.01 – 0.83

SI Dataset S1 (sars-cov-2_and_sars-related_25_genomes_msa_structures.txt)
Complete results from LinearTurboFold for 25 SARS-CoV-2 and SARS-related genomes.

References

1. JH Havgaard, J Gorodkin, RNA structural alignments, part I: Sankoff-based approaches for structural alignments in *RNA Sequence, Structure, and Function: Computational and Bioinformatic Methods.* (Springer), pp. 275–290 (2014).
2. K Asai, M Hamada, RNA structural alignments, part II: non-Sankoff approaches for structural alignments in *RNA Sequence, Structure, and Function: Computational and Bioinformatic Methods.* (Springer), pp. 291–301 (2014).
3. PP Gardner, R Giegerich, A comprehensive comparison of comparative RNA structure prediction approaches. *BMC Bioinforma.* **5**, 1–18 (2004).
4. MS Waterman, Computer analysis of nucleic acid sequences in *Methods in enzymology.* (Elsevier) Vol. 164, pp. 765–793 (1988).
5. SH Bernhart, IL Hofacker, S Will, AR Gruber, PF Stadler, RNAalifold: improved consensus structure prediction for RNA alignments. *BMC Bioinforma.* **9**, 1–13 (2008).
6. MS Waterman, Consensus methods for folding single-stranded nucleic acids. *Math. methods for DNA sequences/editor, Michael S. Waterman* (1989).
7. M Hochsmann, T Toller, R Giegerich, S Kurtz, Local similarity in RNA secondary structures in *Computational Systems Bioinformatics. CSB2003. Proceedings of the 2003 IEEE Bioinformatics Conference. CSB2003.* (IEEE), pp. 159–168 (2003).
8. S Siebert, R Backofen, MARNA: A server for multiple alignment of RNAs. in *German Conference on Bioinformatics.* pp. 135–140 (2003).
9. D Sankoff, Simultaneous solution of the RNA folding, alignment and protosequence problems. *SIAM J. on Appl. Math.* **45**, 810—-825 (1985).
10. S Will, K Reiche, IL Hofacker, PF Stadler, R Backofen, Inferring noncoding RNA families and classes by means of genome-scale structure-based clustering. *PLoS Comput. Biol.* **3**, e65 (2007).
11. JH Havgaard, E Torarinsson, J Gorodkin, Fast pairwise structural RNA alignments by pruning of the dynamical programming matrix. *PLoS Comput. Biol.* **3**, 1896–1908 (2007).
12. Y Tabei, H Kiryu, T Kin, K Asai, A fast structural multiple alignment method for long RNA sequences. *BMC Bioinforma.* **9**, 33 (2008).
13. Z Xu, DH Mathews, Multilign: an algorithm to predict secondary structures conserved in multiple RNA sequences. *Bioinformatics* **27**, 626–632 (2011).
14. DH Mathews, DH Turner, Dynalign: an algorithm for finding the secondary structure common to two RNA sequences. *J. Mol. Biol.* **317**, 191–203 (2002).
15. K Sato, Y Kato, T Akutsu, K Asai, Y Sakakibara, DAFS: simultaneous aligning and folding of RNA sequences via dual decomposition. *Bioinformatics* **28**, 3218–3224 (2012).
16. JN Zadeh, BR Wolfe, NA Pierce, Nucleic acid sequence design via efficient ensemble defect optimization. *J. Comput. Chem.* **32**, 439–452 (2010).
17. K Katoh, DM Standley, MAFFT multiple sequence alignment software version 7: improvements in performance and usability. *Mol. Biol. Evol.* **30**, 772–780 (2013).
18. R Rangan, et al., De novo 3D models of SARS-CoV-2 RNA elements from consensus experimental secondary structures. *Nucleic Acids Res.* **49**, 3092–3108 (2021).
19. O Ziv, et al., The short- and long-range RNA-RNA interactome of SARS-CoV-2. *Mol. cell* **80**, 1067–1077 (2020).
20. R Rangan, et al., RNA genome conservation and secondary structure in SARS-CoV-2 and SARS-related viruses: a first look. *RNA* **26**, 937–959 (2020).
21. NC Huston, et al., Comprehensive in vivo secondary structure of the SARS-CoV-2 genome reveals novel regulatory motifs and mechanisms. *Mol. cell* **81**, 584–598 (2021).
22. A Afzal, Molecular diagnostic technologies for COVID-19: Limitations and challenges. *J. Adv. Res.* (2020).
